# Development of a set of patient-centred outcome measures for patients with major injury: Delphi-based consensus recommendations from the International Consortium for Health Outcomes Measurement Major Injury Working Group

**DOI:** 10.1016/j.eclinm.2025.103617

**Published:** 2025-11-03

**Authors:** Henk van der Wal, Winny Collot d’Escury, Umanga de Silva, Yasmine Saoud, Grace Jennings, Leanne M. Aitken, Katelyn Carey, Sharfuddin Chowdhury, Ian Civil, Damian Clarke, Tim Cudmore, Candi Diaz, Juan P. Herrera-Escobar, Rigo Hoencamp, Karen Hoffman, Kelly Lang, Katherine Martin, Debra Marvel, Joseph Mathew, Emer McGilloway, Stefaan Nijs, Wellingson Silva Paiva, Kat Quick, Marcelo A.F. Ribeiro, Therese S. Richmond, Gunnar Sandersjöö, Takeshi Sawaguchi, Martin Schreiber, Michael Schuetz, Hilaire Thompson, Thijs van Dongen, Remko van Lutterveld, Guixi Zhang, Belinda Gabbe

**Affiliations:** a Trauma Research Unit, Department of Surgery, Erasmus MC, University Medical Center Rotterdam, Rotterdam, the Netherlands; b Defence Healthcare Organization, Ministry of Defence, Utrecht, the Netherlands; c Leiden University Medical Center, Leiden, the Netherlands; d International Consortium for Health Outcomes Measurement, Boston, MA, USA; e City St George's, University of London, London, UK; f Beauty After Breast Cancer, NH, USA; g King Saud Medical City, Riyadh, Saudi Arabia; h University of Auckland, New Zealand; i University of KwaZulu-Natal, Durban, South Africa; j University of the Witwatersrand, Johannesburg, South Africa; k Cudmore Consulting, Australia; l Coalition for National Trauma Research, TX, USA; m Harvard Medical School—Brigham and Women's Hospital, MA, USA; n Queen Mary University of London, UK; o The Royal Melbourne Hospital, Australia; p Department of Surgery, Monash University, National Trauma Research Institute, Melbourne, Victoria, Australia; q Trauma Services, Alfred Health, Melbourne, Victoria, Australia; r King's College Hospital London, Cleveland Clinic, London, UK; s University Medical Center Utrecht, Utrecht, the Netherlands; t University of São Paulo, Brazil; u Te Tāhū Hauora Health Quality and Safety Commission, New Zealand; v University of Maryland School of Medicine—R Adams Cowley Shock Trauma Center, Baltimore, MD, USA; w University of Pennsylvania School of Nursing, Leonard Davis Institute of Health Economics, Penn Injury Science Center, PA, USA; x Karolinska University, Stockholm, Sweden; y Fukushima Medical University, Shinyurigaoka General Hospital, FFN Japan, Japan; z Department of Surgery, Uniformed Services University, MD, USA; aa Jamieson Trauma Institute, Royal Brisbane and Women's Hospital, Metro North Health, Brisbane, QLD, Australia; ab Queensland University of Technology, Brisbane, QLD, Australia; ac University of Washington School of Nursing—Harborview Injury Prevention and Research Center Seattle, WA, USA; ad Brain Research and Innovation Center, Ministry of Defence, Utrecht, the Netherlands; ae Department of Psychiatry, University Medical Center Utrecht, the Netherlands; af Department of Surgery, School of Medicine, Chinese University of Hong Kong, Shenzhen, China; ag School of Public Health and Preventive Medicine, Monash University, Australia; ah Population Data Science Swansea University Medical School, Swansea University, UK

**Keywords:** Major injury, Value-based-healthcare, Delphi study, Patient-reported outcome, PROM

## Abstract

**Background:**

Major injury contributes substantially to global morbidity and mortality, yet outcome measurement remains inconsistent. Development of a standardised set of patient-centred outcome measures for adults with major injuries has the potential to improve global benchmarking, quality of care and patient-centre value-based healthcare.

**Methods:**

An international multidisciplinary working group of 28 experts and patient representatives from 11 countries (3 middle- and 8 high income) was convened. Following International Consortium for Health Outcomes Measurement methodology, a structured consensus-driven process was used, which included literature reviews, three modified Delphi rounds, and validation surveys. The target population included adult patients receiving acute care for physical injuries with an Injury Severity Score ≥9. Literature searches (Apr 16, 2005–Jun 1, 2024) in PubMed, MEDLINE, Embase.com, PsychINFO, CINAHL, and ProQuest identified relevant outcomes, measures, case-mix variables, and follow-up timepoints. Consensus on inclusion required ≥80% of members rating outcomes 7–9 on a 9-point scale. The Delphi process ran from Jul 3 to Nov 13, 2024, with patient and professional validation surveys respectively conducted between Dec 5, 2024 and Mar 28, 2025 and between Mar 5, and 28, 2025.

**Findings:**

Seventy-two percent (n = 20) of members participated in eight calls, and 68% (n = 19) in nine surveys. The final set includes 26 outcomes (22 patient-reported, 4 clinician-reported) across four domains: patient-reported health status, functioning, psychological wellbeing and mental health, and clinical outcomes. Six patient-reported outcome measures and four injury-specific tools were endorsed. Thirty case-mix variables were identified, with measurement at baseline (as soon as possible after the injury, conditional upon the clinical stability of and safety for the patient), 6 months, and 12 months. Validation surveys with patients (n = 121) and professionals (n = 70) confirmed relevance and comprehensiveness, with >83% (n = 159) agreement on core domains. A primary subset of essential outcome measures was defined to facilitate implementation.

**Interpretation:**

To our knowledge, this is the first international consensus-derived outcome set for major injury, supporting benchmarking of care, of outcomes of importance to patients and clinicians. We acknowledge the lack of representation from low-resource countries or regions in the working group and patient survey. Further implementation and feasibility testing are needed to ensure applicability across diverse populations, health systems and settings (e.g. military). In particular, validation in low- and middle-income settings is needed to ensure equity, feasibility and cultural relevance.

**Funding:**

10.13039/501100001250Transport Accident Commission, Stichting ZiektekostenVerzekering Krijgsmacht, 10.13039/501100001702AO Foundation.

**Research in context**

**Evidence before this study**

Before undertaking this project, we reviewed published evidence on consensus-based efforts to define outcome measures in trauma and related fields. To identify relevant studies, we examined PubMed/MEDLINE, Embase, PsycINFO, CINAHL, ProQuest, and reference lists of key journals and reports, covering publications from Apr 16, 2005 to Jun 1, 2024 without language restrictions. Search terms combined concepts for trauma, major injury, polytrauma, core outcome set, Delphi consensus and patient-reported outcomes. The most relevant initiative was the U.S. National Trauma Research Action Plan (NTRAP), which used a Delphi process to define core outcome domains for long-term trauma research. However, NTRAP emphasised research endpoints rather than standardised international clinical outcome sets. Other consensus-based frameworks have addressed specific injuries—such as traumatic brain injury, spinal cord injury, or burns—or adjacent fields including hand/wrist conditions, osteoarthritis, obesity, and low-back pain. These efforts showed Delphi's feasibility, sometimes with patient input, yet none captured the full spectrum of major injury across mechanisms, severities, and global health system contexts.

The quality of the available evidence was moderate. Existing studies generally followed rigorous consensus methods but were geographically limited (predominantly North America and Europe) and often excluded patient perspectives or settings outside of high-income countries. No meta-analyses or pooled estimates of trauma outcome domains were available, and there remained a lack of globally applicable, patient-centred consensus guidance for major injury.

**Added value of this study**

This study is the first to develop an international, consensus-based, patient-centred outcome set for adults with major injury. Using the structured methodology of the International Consortium for Health Outcomes Measurement (ICHOM), we combined expert and patient perspectives from 11 countries. The process yielded 26 outcomes and 30 case-mix variables, along with validated measurement tools and harmonised follow-up time points. Importantly, the set was refined through international validation surveys with patients and professionals, ensuring both clinical and patient relevance.

**Implications of all the available evidence**

Taken together, previous Delphi initiatives and our findings demonstrate that while outcome consensus work has advanced in specific domains, a unified, internationally applicable framework for major injury outcomes was lacking. The outcome set developed here addresses this critical gap, enabling consistent measurement of outcomes that matter most to patients, supporting global benchmarking, and promoting value-based trauma care. However, we acknowledge as a key limitation the limited representation from low-resource countries in both the working group as patient and health professional surveys, which may restrict the global applicability of the findings. Future research should focus on evaluating the feasibility, acceptability, and impact of implementing this set in real-world clinical practice. This also requires validation in low- and middle-income countries to ensure equity and cultural relevance.

## Introduction

Traumatic (physical) injury accounts for 4·4 million deaths annually and comprises 8% of global mortality.^1^ Each year, tens of millions of people survive their injuries, leading to a substantial burden on healthcare.^1^^,^^2^ Global forecasts indicate rising life expectancy, with a continued shift from communicable diseases to non-communicable diseases, including injury. A recent Global Burden of Disease study has predicted a rise in the proportion of disability-adjusted life years attributed to years lived with disability increasing from 33·8% to 41·1% by 2050.^3^

The severity of injury ranges from minor, which typically involves isolated or single system injuries without threat to life or disability, to major, which can involve complex injuries to single or multiple systems (polytrauma or multi-trauma). Major injuries produce diverse and far-reaching effects, with many survivors experiencing long-term or permanent disability requiring extensive physical and psychological rehabilitation.^4^ Major injury exerts a multidimensional impact on overall physical health, impacting on financial stability and return to work, as well as physical functioning (fatigue, sleep, sexual functioning, etc.).^5^ Post-traumatic stress, depression, and anxiety are increasingly recognised as important sequelae of major injury, with recommendations made for early referral and intervention to reduce the risk of worse long term patient outcomes.^6^

Given the prevalence of long-term health and social impacts of major injury, it is imperative to increase emphasis on the evaluation of outcomes in order to obtain a comprehensive understanding of recovery patterns and trajectories (e.g. who, when, and how well people recover from major injury), inform improvements in healthcare, monitor the impacts of policy and practice change, benchmark trauma care, and support value-based trauma care.^7^ Value-based Healthcare (VBHC), a concept introduced in 2006 by Porter and Teisberg, is a model of delivering high-value care for patients.^8^ Value in healthcare is measured through the outcomes achieved, not by the volume of services delivered.^9^ It prioritises the health outcomes that matter most for the patient as the foundation of value.

In recent years, several studies have focused on establishing frameworks for measuring injury outcomes.^10^^,^^11^ Notably, the National Trauma Research Action Plan (NTRAP) project undertook a comprehensive approach to define optimal metrics to assess long-term outcomes following hospital discharge.^12^ Existing studies and frameworks have predominantly focused on high-income countries (HIC).^13^^,^^14^ Differences in culture, resource availability across countries, and the challenges of outcome measurement in conflict-affected regions warrant consideration in the development of a global outcome set for major injury.

The International Consortium for Health Outcomes Measurement (ICHOM) is a not-for-profit organisation that has developed 47 sets of patient-centred outcome measures for diverse medical conditions. To understand the outcomes that matter for patients with major injuries and to enhance care through a value-based healthcare approach, ICHOM assembled a globally diverse working group with clinicians, researchers, and patient representatives. The aim of this initiative was to define a globally applicable set of patient-centred outcome measures and case-mix factors for major injury patients to facilitate the evaluation, benchmarking, and improvement of clinical care for patients with major injuries, focussing on outcomes that matter most to the patient.

## Methods

### Design and development of the major injury (MJI) set

A multidisciplinary, geographically dispersed, working group of professionals and patient representatives was assembled through an invitation-based selection process using snowball sampling.^15^ Coordinated by the project team, both its own networks, and those of the working group members, were used to promote broad global representation, with balanced participation from low-, middle-, and high-income countries, and equitable inclusion of patient representatives. Members included clinical and research experts in trauma surgery, trauma nursing, patient advocacy, public health, rehabilitation, and outcomes measurement. Several of whom had prior experience developing national outcomes and implementation pathways. Thirty members were recruited from 11 countries: Australia, Brazil, China, Japan, the Netherlands, New Zealand, Saudi Arabia, South Africa, Sweden, the United Kingdom, and the United States. The sample size was determined pragmatically in accordance with ICHOM methodology, prioritising multidisciplinary diversity and global representation over statistical power. The final group of 28 experts and patient representatives from 11 countries (three middle- and eight high-income) provided sufficient diversity for structured consensus building across three Delphi rounds. This aligns with established consensus methods recommending panels of 20–30 participants to achieve content validity and stable agreement.^16^ Open validation surveys were subsequently used to capture a broad range of professional and patient perspectives from different regions to assess face and content validity.

**Fig. 1:**
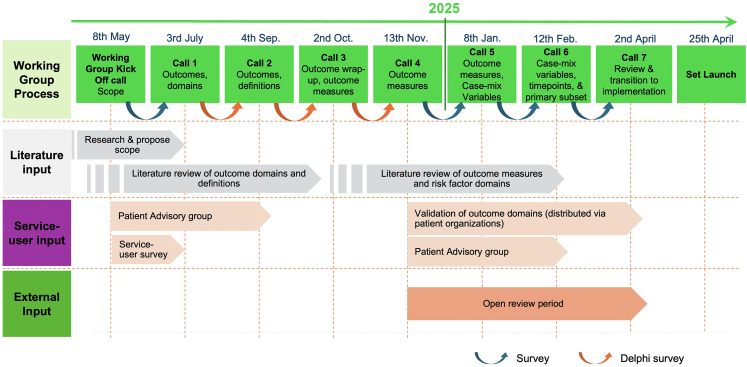
Overview of stages of the Major Injury Set development process. The figure presents a timeline of the Major Injury Set development process between May 2024 and April 2025, outlining the stages from the initial scope definition to the set launch. It illustrates the integration of working group calls, literature input, service-user contributions, and external review, supported by surveys (blue arrow) and a three-round Delphi process (orange arrow).

Development followed the structured ICHOM methodology (Supplementary material S1 Detailed methodology), as per previously published sets,^17^, ^18^, ^19^ and followed The Revised Standards for Quality Improvement Reporting Excellence (SQUIRE 2.0) guidelines.^20^ The methods comprised research data obtained through systematic literature searches, expert opinion and lived experience. Decision-making was conducted through a working group discussions and modified Delphi surveys (Fig. 1). Working group membership and authorship eligibility were based on meeting minimum participation criteria, including active working group membership, attendance at ≥50% of meetings (with the option to provide offline feedback for missed sessions), completion of ≥50% of surveys, and review and feedback on the manuscript, in accordance with ICMJE authorship requirements. These criteria were presented at the kick-off meeting and reiterated during subsequent meetings to ensure transparency and consistency.

Set scope was established through consensus at the kick-off meeting. Subsequent MJI Working Group (MJIWG) online meetings established the relevant domains, outcomes, outcome measures, case-mix variables, and time points (Fig. 1). Patient representatives also participated in the Patient Advisory Group (PAG), a subgroup of the MJIWG. A structured, consensus-driven, modified three-round Delphi methodology was employed. Finally, external validation took place through patient and professional validation surveys, incorporating feedback from patients, experts, and other relevant stakeholders. A project team provided logistical and strategic support for the MJIWG.

### Defining the scope

The first step was to define the applicable patient population. Based on a review of the literature, the project team drafted a scope for the MJI set. This draft was presented to the MJIWG for discussion and amendment prior to a vote on agreement.

### Modified Delphi process

**Fig. 2:**
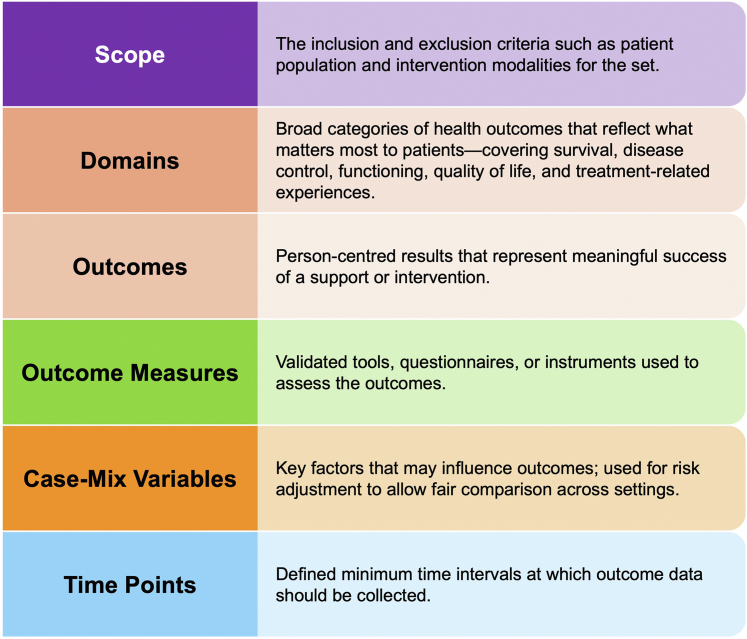
Elements for set definition. The figure outlines six essential elements for a standardised set of patient-centred outcome measures: Scope, Domains, Outcomes, Outcome Measures, Case-Mix Variables, and Time Points.

Literature review findings relevant to the set elements (Fig. 2) were first presented to the MJIWG for discussion and suggestions. Following the meeting, members were required to vote on each item in an online Modified Delphi process using a 9-item Likert scale (1 = least essential, 9 = most essential). The results of each round were discussed during the next meeting. To achieve inclusion in the first Delphi round, ≧80% of the MJIWG had to score an item between ‘7’ and ‘9’ (between essential and most essential). Items with ≧80% consensus response of a score between ‘1’ and ‘3’ were immediately excluded. All others were considered inconclusive and progressed to the next voting round. The same approach was used for the second Delphi round. The third Delphi round included inconclusive items from the second round. Respondents were provided with the options ‘include’ or ‘exclude’ and ≧70% was considered consensus. A 70% consensus threshold aligning with established methods for inclusion of items with broad expert agreement. Additionally, potential overlaps between respective items were examined to determine whether outcomes could be merged. The Delphi rounds were conducted between Jul 3 and Nov 13, 2024 (Fig. 1).

### Search strategy and selection criteria

A comprehensive literature search was conducted, identifying potentially relevant clinical outcomes, patient-reported outcomes, case-mix variables, and time points for collection from published studies and clinical guidelines between Mar 1, 2005, and May 31, 2024. Two investigators (HVDW, WCD) conducted the search using PubMed, MEDLINE, Embase.com, PsychINFO, CINAHL, and ProQuest. The final search was performed on Jun 13, 2024. The full search strategies are provided in Supplementary material S2. Articles were exported to Covidence.^21^ The project team (HVDW, WCD, UD, YS) independently screened selected articles for eligibility. Studies were eligible if they reported clinical and/or patient-reported outcomes in adults (≥18 years) hospitalised for traumatic injury, including blunt force trauma, penetrating trauma, geriatric trauma, self-inflicted injury, combat-related injury, or traumatic brain injury. Only studies using validated patient-reported outcome tools and providing an English-language abstract were included. We excluded studies focussing on paediatric populations (<18 years), those with insufficient information on outcome definitions or unclear diagnoses, and studies using non-validated patient-reported outcome (PRO) tools. Questionnaires administered to the general public or carers, rather than to patients living with the consequences of traumatic injury, were also excluded. Disagreements between reviewers were discussed and resolved by the project team. Subsequently, relevant data points were extracted and organised into Google Sheets for data management.

### Identification and selection of domains and outcomes

Given the large number of search results, articles were randomly selected for abstract screening, consistent with ICHOM methods.^19^^,^^22^ All references were randomised by assigning each a unique random identifier, after which they were sorted accordingly. Articles were then selected following this randomised sequence. Abstracts were screened by four team members (HVDW, WCD, UD, YS) based on predefined eligibility criteria. A random sample of 500 articles was repeatedly selected and screened from the results, applying a saturation method. As long as new relevant findings continued to emerge, an additional 500 articles were screened. The objective was saturation of results while targeting at least 125 articles for full-text screening. Domains and domain-related outcomes were extracted from the full-text screening and presented to the MJIWG and PAG for discussion who provided input on any potential missing set items deemed important to include in the first round of the Delphi process, ensuring consideration of the priorities of patients and healthcare professionals.

### Identification and selection of measures

The project team reviewed the literature on patient-reported outcome measures (PROMs) and clinician-reported outcome measures (CROMs) for the various outcomes. Depending on the search strategy result volume, all or a random selection of 250 abstracts were screened. Relevant PROMs and CROMs were extracted and underwent psychometric property analysis, including the number of translations, costs, number of questions, completion time, age applicability, sensitivity to change, content validity, construct validity, discriminative validity, test-retest reliability, and internal consistency. A comprehensive overview of these assessments was presented to the MJIWG to inform the selection process.

### Identification and selection of case-mix variables

The project team selected relevant case-mix variables from literature searches, suggestions from MJIWG members, and ICHOM harmonised case-mix variables from existing sets. Case-mix variable inclusion was evaluated according to: (1) relevance (strength of causal linkage between the case-mix variable and the outcome), (2) independence (improves predictive power over variables already included), (3) practicality of measurement (not harmful, expensive, or uncomfortable), and (4) the feasibility for international comparison. These case-mix variables were subsequently presented to the MJIWG for decision.

### Identification and selection of time points

Time points for collection of each recommended outcome and measurement were derived from the articles reviewed in the preceding steps. The project team presented the results of a structured framework for data collection, which was subsequently considered by the MJIWG.

### Open review process

An open review process conducted prior to the launch of the final set consisted of patient and professional validation surveys administered through the Qualtrics survey platform. The aim of these surveys was to establish whether the set developed by the MJIWG included outcomes and case-mix variables of relevance and importance to people with lived experience of, and professionals involved in the care of, major injury. The patient survey was administered in Australia, the Netherlands, New Zealand, United Kingdom, and the United States from Dec 5, 2024 to Mar 28, 2025. Participants received, as part of the overall questionnaire, an informed consent form, and a brief introduction to VBHC and the study before completing the questionnaire. Ethics approval or exemption was obtained from ethics committees and regulatory bodies in each country (see Supplementary material S3). The anonymous professional survey was available internationally from the Mar 5 to Mar 28, 2025, in 16 countries (Australia, New Zealand, United States, United Kingdom, Germany, India, Brazil, Canada, Ethiopia, Malaysia, the Netherlands, France, Norway, Philippines, Saudi Arabia, and South Africa). Surveys were widely distributed to the ICHOM network and the professional network of the MJIWG members. Patient participants were only asked for feedback on the outcomes, while the professional participants were asked for feedback on the entire developed set (outcomes, outcome measures, case-mix variables, and time-points). The findings were discussed at the final MJIWG meeting and considered in defining the final set. Ethics approval or exemption was obtained where required, including for the patient survey. Formal approval was not mandatory for the Delphi and professional open review surveys given the study design. Participation was voluntary and anonymous, and based on electronic informed consent.

### Primary subset

A primary subset of essential outcome measures was defined to help implementers begin measuring outcomes that matters most to major injury patients. This subset was drawn from the comprehensive core outcome set through a final voting process by the MJIWG. Members ranked 26 outcomes on a 9-point Likert scale (1 = Not important 5 = Somewhat important 9 = Most important), with inclusion requiring ≥80% agreement at a score of 7 or higher, or ≥70% for other variables selected based on clinical implementation feasibility. Furthermore, case-mix variables that were ratified in the final set were incorporated into the primary subset to facilitate downstream data collection and comparison. The process ensured that the primary subset represented both clinical importance and practical applicability across diverse settings, regardless of geography or resources. After voting, the MJIWG reviewed and discussed feasibility, confirming that the selected measures could serve as a universal foundation for consistent and meaningful outcome measurement in major injury care.

### Role of the funding source

Study funders had no role in study design, data collection, data analysis, data interpretation, or writing of the report. All authors (including the MJIWG members) had access to the underlying data, reviewed and approved the final version of the manuscript. BG, HVDW, UD, and GJ had final responsibility for the decision to submit for publication.

## Results

On average, 72% (n = 20) of the 28 MJIWG members participated in each of the eight calls; 68% (n = 19) responded to each of the nine surveys. Two members (7%) of the original 30 MJIWG members participated in <50% of calls and surveys and were excluded from the final list, according to the ICHOM project authorship criteria. The baseline demographic characteristics of the members of the MJIWG are provided in the Supplementary material S4.

### Scope for the Major Injury Set

**Fig. 3:**
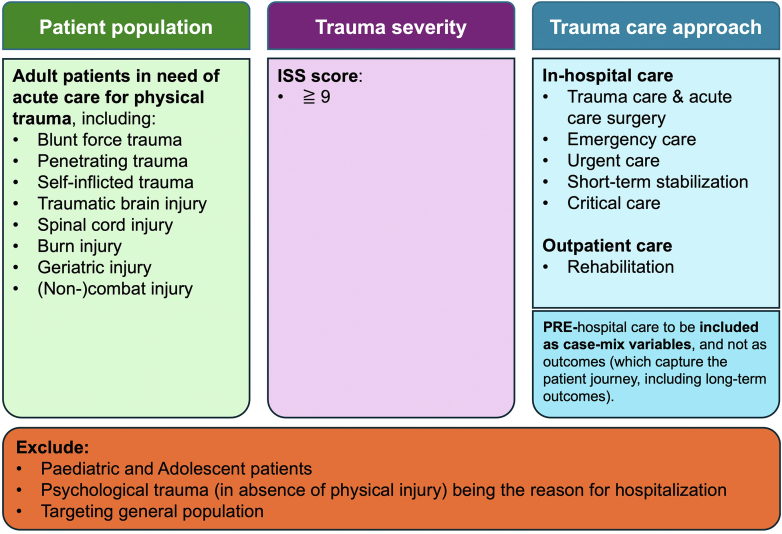
Scope of the Major Injury Set. The figure defines the scope of the Major Injury Set by outlining inclusion criteria based on patient population (adult patients requiring acute care for various forms of physical trauma), trauma severity (Injury Severity Score (ISS) ≥ 9), trauma care approach and the exclusion criteria.

During the kick-off call, a decision tree with the elements of age, trauma severity, and trauma mechanism was used to discuss the draft scope. Based on the discussion, the MJIWG voted on the proposed scope in the post-call survey; 84% (n = 24) agreed and the scope was ratified at the next MJIWG call. The MJIWG defined the scope as adult patients requiring acute care for physical injuries with an Injury Severity Score (ISS) of ≥9, including blunt force trauma, penetrating trauma, self-inflicted trauma, geriatric trauma, combat and non-combat injuries, traumatic brain injury (TBI), spinal cord injury (SCI), and burn injury (Fig. 3).

The MJIWG chose to focus on adults. Recognising that the cut-off age for an ‘adult’ varies globally, the MJIWG agreed to support the use of locally defined cut-off ages for adults as a starting point for this set. Furthermore, it was determined that a single set would not adequately address both physical and psychological injury. Thus, patients admitted due to psychological trauma in the absence of physical injury were excluded from this set. Finally, for a major injury care approach, the focus was on in-hospital care and the rehabilitation and recovery phase. Wide variability in pre-hospital care models across countries was considered a significant challenge for collection of data for a global outcomes set. Finally, for a major injury care approach, the focus was on in-hospital care and rehabilitation care.

### Outcome domains and outcomes

**Fig. 4:**
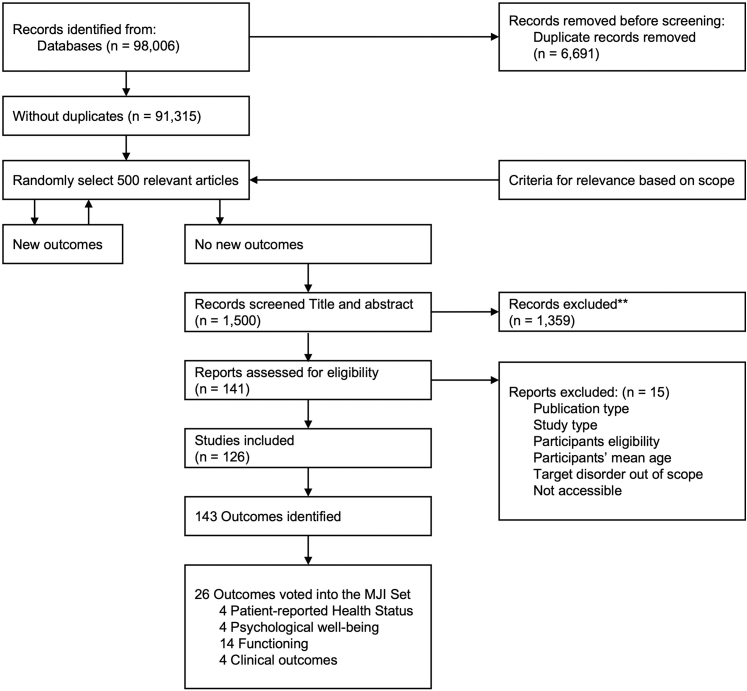
Flowchart of search strategy for outcomes. The flowchart illustrates the systematic search strategy used to identify relevant outcomes for the Major Injury (MJI) Set, starting with 91,315 unique records. Following title and abstract screening (n = 1500) and eligibility assessment (n = 141), 126 studies were included, resulting in 143 outcomes, of which 26 were voted into the final MJI Set.

An extensive search strategy was employed and 91,315 records identified (Fig. 4). To confirm the pre-defined/-selected outcomes using randomisation and saturation, 1500 were screened based on title and abstract; 126 were deemed eligible for inclusion. Full-text screening identified 143 potential outcomes. To reach consensus, three Delphi rounds were conducted, initially resulting in 26 outcomes, 22 PROs and four Clinician-Reported Outcomes (CROs) (Table 1).

**Table 1:** Domains, outcomes and outcome measures of the Major Injury Set.

Domain	Outcome	EQ-5D-5L PROMIS GH-10 VR-12 SF-12	ASSIST-LITE	PC-PTSD	PROMIS sleep disturbance 8b	PROMIS satisfaction with sex life
Patient Reported Health Status	Symptom Severity	+	−	−	−	−
	Pain	+	−	−	−	−
	Disability	+	−	−	−	−
	Health Related Quality of Life	+	−	−	−	−
Psychological Well-being & Mental Health	Substance Use	−	+	−	−	−
	Post-traumatic stress disorder	−	−	+	−	−
	Depression	+	−	−	−	−
	Anxiety	+	−	−	−	−
Functioning	Overall Functioning^b^	+	−	−	−	−
	Physical Health	+	−	−	−	−
	Global Functioning	+	−	−	−	−
	Physical Functioning	+	−	−	−	−
	Musculoskeletal Functioning	+	−	−	−	−
	Ambulatory Functioning	+	−	−	−	−
	Social Functioning	+	−	−	−	−
	Activities of Daily Living	+	−	−	−	−
	Autonomy	+	−	−	−	−
	Occupational Functioning	+	−	−	−	−
	Changes in Employment	+	−	−	−	−
	Sexual Function	−	−	−	−	+
	Sleep	−	−	−	+	−
	Cognitive Functioning^a^	−	−	−	−	−
Clinical Outcomes^c^	Mortality	−	−	−	−	−
	Complications	−	−	−	−	−
	Discharge Location	−	−	−	−	−
	Ongoing Injury Management	−	−	−	−	−

a Reported using MPAI-4 for patients with head injuries.

b Injury-specific functioning additionally reported for relevant patients using MPAI-4 (TBI), SCIM-SR (SCI), and BSHS-B (burns).

c Reported using clinician-reported outcome measures (CROMs).

All four outcome domains initially selected by the MJIWG were rated as essential by >70% (n = 85) of patient-validation survey respondents. 100% (n = 4) of outcomes in the Patient-Reported Health Status domain, 100% (n = 14) of outcomes in the Functioning domain, and 80% (n = 3) of outcomes in the Psychological Well-being & Mental Health domain were considered most important by the respondents. While the substance use outcome was not considered most important by the patient validation survey respondents, the PAG considered it essential to the set. For the Clinical Outcomes domain, 50% (n = 2) of outcomes were considered important by all respondents. Across all domains, 87% (n = 105) of respondents agreed that the most important outcome areas relevant to patients had been successfully identified. Additionally, 97% (n = 117) indicated that the availability of such a set would be valuable. There was universal endorsement of the set by the professional validation survey respondents; 89% (n = 62) of participants agreed with the proposed outcomes, case-mix variables, and time points and 86% (n = 60) agreed that no critical concepts were missed.

### Outcome measures

A total of 1782 articles were screened and 299 measurement tools identified, of which, the most frequently cited (n = 29) proceeded to psychometric assessment through published validation studies conducted in major injury populations, or, if unavailable, in the general population. The psychometric properties, including the time to complete, of the selected PROMS are provided in the Supplementary material S5. The findings were presented to the MJIWG and evaluated using the modified Delphi process.

The final set includes up to six PROMs, depending on type of MJI (Table 1). This approach ensures comprehensiveness while minimising administrative and patient burden. A crosswalk approach was ratified for the EQ-5D-5L, PROMIS GH-10, VR-12, and SF-12 allowing users of the set to select one of these PROMs based on organisational/personal preference or experience. The crosswalk instruments cover outcomes within the Patient-Reported Health Status domain, depression and anxiety within the Psychological Well-being & Mental Health domain, and overall functioning, physical functioning, social functioning, and occupational functioning within the Functioning domain. The ASSIST-LITE measures substance use and the PC-PTSD measures post-traumatic stress disorder (PTSD). Injury-specific outcome measures are included to cover overall functioning for TBI (MPAI-4), SCI (SCIM-SR), and burns (BSHS-B). The PROMIS Sleep Disturbance 8b and Satisfaction with Sex Life scales are included to measure sleep and sexual functioning, respectively. For patients with TBI, cognitive functioning is measured using the MPAI-4.

### Case-mix variables

The MJIWG identified case-mix variables that are useful and minimise burden on the patient and healthcare professional. The application of case-mix variables, in addition to the ICHOM variables used, is supported by: (1) Self-Administered Comorbidity Questionnaire (SACQ) for comorbidities, (2) Injury Severity Score (ISS) for injury severity, and (3) Glasgow Coma Scale (GCS) for level of consciousness. The case-mix variables included in the set (Table 2) are categorised into three groups: (1) demographic, (2) baseline health status, clinical, and injury event, and (3) treatment-related factors. Further detail and how to use the case-mix variables are provided in the reference guide.^23^

**Table 2:** Case-mix variable domains and definitions.

Case-mix category/variable	Definition (response options)	Reported
Demographic factors		
Age	Year of birth	Professional
Sex	The patient's sex at birth	Professional
Gender identity	The patient's gender identity^a^	Patient
Level of education	Highest level of education completed based on local standard definitions of education levels^b^	Patient
Race	The biological race of the person^c^	Patient
Ethnicity	The cultural ethnicity of the person that they most closely identify with^c^	Patient
Work status	What is your work status?	Patient
Work status/Educational status	The patient's work/educational status	Patient
Financial resources	How hard is it for you to pay for the very basics like food, housing, medical care, and heating?	Patient
Relationship status	A person's current relationship status	Patient
Social Support	How many people do you have near you that you can readily count on for help in time of difficulty such as to watch over children or pets, give rides to the hospital or store, or help when you are sick?	Patient
Occupation	What is your occupation?	Patient
Baseline Health Status, Clinical, and Injury Event Factors		
Body mass index	Body mass index	Professional
SACQ comorbidities	Indicate whether the patient has a documented history of any of the following comorbidities: heart disease, high blood pressure, lung disease, diabetes, ulcer or stomach disease, kidney disease, liver disease, anaemia or other blood disease, cancer/other cancer, depression, osteoarthritis/degenerative arthritis, back pain, rheumatoid arthritis, or other medical problems a patient is experiencing.	Patient
	Indicate if any of the reported comorbidities limits their function	Patient
	Please indicate the summed score for all of the patient's comorbidities, with a maximum of 3 points for each medical condition.	Professional
Psychiatric comorbidities	Have you ever been told by a doctor that you have any of the following (select all that apply): I have no other mental health conditions, or social anxiety/phobia, generalised anxiety, OCD, panic disorder, agoraphobia, habit problems, depression, self-injury/self-hard, bipolar disorder, psychosis, substance abuse, ADHD/hyperactivity, poses risk to other, PTSD, family relationship difficulties, persistent difficulties managing relationships with others, gender dysphoria, unexplained physical symptoms, self-care issues, adjustment to health issues, and other issues.	Patient
Smoking status	A person's current and past smoking behaviour	Patient
Alcohol Amount per drinking occasion	On average, how many units of alcohol does the patient consume per occasion of drinking alcoholic drinks or beverages?	Patient
Type of injury	Which injury type(s) did the patient sustain? (select all that apply)	Patient
Mechanism of injury	What is the mechanism of injury?	Patient
Intent of injury	What was the intent of the injury?	Patient
Context of injury	What are the circumstances and events that led to the context in which the injury occurred?	Patient
ISS^d^	Indicating maximal AIS in regions: head and neck, face, chest, abdomen, extremity (including pelvis), and external region	Professional
GCS	What are the patient's eye-opening, verbal, and motor responses?	Professional
Time from Injury	How much time has passed since the injury occurred?	Professional
Physiologic variables at first medical contact: Heart rate	Indicate the first measurement or earliest record of heart rate (in beats per minute) for this episode of care	Professional
Systolic blood pressure	Systolic reading of blood pressure, as measured by the indicated device type, in mmHg	Professional
Shock Index	Indicate the shock index value, calculated as Shock Index = Heart Rate/Systolic Blood Pressure	Professional
Treatment-Related Factors		
Treatment Type	Did the patient receive one of the listed procedures?	Professional
Prehospital Treatment	What level of pre-hospital treatment was provided to the patient?	Professional
Level of Definitive Care	What is the level of definitive care received by the patient?	Professional
Date of admission to hospital	Indicate the date the patient was admitted	Professional
Date of discharge	Indicate the date the patient was discharged	Professional

SACQ = Self-administered Comorbidity Questionnaire; ISS = Injury Severity Score; GCS = Glasgow Coma Scale; AIS = Abbreviated Injury Scale.

a Sex and gender information are reported using the SAGER guidelines.

b This measure may vary based on local standards for education levels. It is recommended to consult the International Standard Classification of Education.

c This measure should be recorded based on local standards in the particular geographic region and should be voluntarily self-reported by the patient. This optional question is as racially and ethnically inclusive as possible.

d The ISS is an overall measure, which has to be supported by variables like type of injury, to able to define the injury pattern.

### Time points

**Fig. 5:**
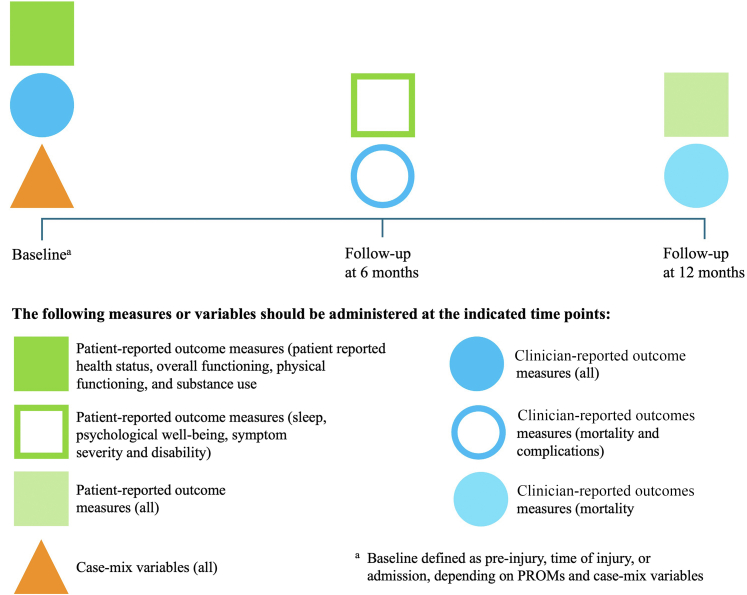
Data collection time points. The figure presents a timeline for data collection at baseline, 6 months, and 12 months, indicating the types of outcome measures to be administered at each point. Legend: dark green square: patient-reported outcome measures (patient-reported health status, overall functioning, physical functioning, substance use), outlined square: patient-reported outcome measures (PROMs) (sleep, psychological well-being, symptom severity and disability), light green square: patient-reported outcome measures (all), dark blue circle: clinician-reported outcome measures (all), outlined circle: clinician-reported outcome measures (mortality and complications), light blue circle: clinician-reported outcome measures (mortality), triangle: case-mix variables (all).

Following literature review results, open review survey input, and extensive MJIWG discussions, consensus on the timing of data collection was reached (Fig. 5). The overall response rate on time point consensus was 82% (n = 23) for the MJIWG and 100% (n = 70) for the health professionals in the open review survey. Consensus within the MJIWG showed greater variability on the time points per respective outcome. For the baseline time point, consensus ranged from 70% to 100% (n = 20 to n = 28); for the 6 months follow-up time point, from 70% to 83% (n = 20 to n = 23); and for the 1-year follow-up time point, from 74% to 91% (n = 21 to n = 25). In contrast, the open review health professional's consensus was consistently high across all timepoints, with 91% (n = 64) agreement for the baseline time point, 90% (n = 63) for the 6 months follow-up, and 95% (n = 67) for the 1-year follow-up measure. Five (7%) of the open review health professionals agreed with the time-point, but also provided a proposal to modify the time points. These proposals largely aligned with the discussions held within the MJIWG. They included the desirability and feasibility of measuring outcomes annually after the 1-year follow-up. Both groups indicated that it would remain challenging to measure outcomes at baseline and at later follow-ups, especially from the 2-year follow-up onwards. The timing of data collection was approached from the acute setting in which the patient with major injury enters the care pathway. Baseline is ideally defined as the pre-injury status; however, given the unpredictable nature of major injury, baseline outcome measurements are performed as soon as possible after the injury, conditional upon the clinical stability of and safety for the patient. The MJIWG reached a consensus for three-time points; baseline, 6 months, and 1 year. Case-mix variables are measured at baseline.

### Patient experts and health professionals open review

**Fig. 6:**
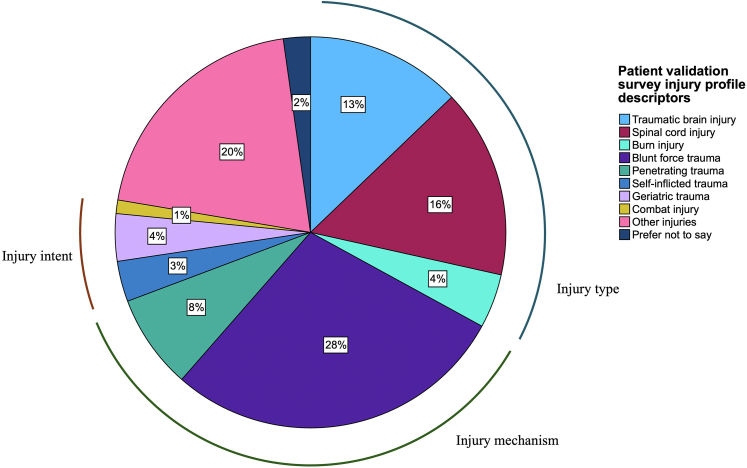
Multiple injury profile descriptors, distribution among patient-validation survey group. The pie chart displays the distribution of multiple injury profile descriptors among the patient-validation survey group, categorised into injury type, mechanism, and intent. The largest proportion is attributed to blunt force trauma (28%), followed by burn injury (20%) and spinal cord injury (16%). Legend: light blue: traumatic brain injury, burgundy: spinal cord injury, turquoise: burn injury, purple: blunt force trauma, green: penetrating trauma, blue: self-inflicted trauma, lavender: geriatric trauma, yellow: combat injury, pink: other injuries, dark blue: prefer not to say.

A total of 121 participants (in the age range from 18 to 85 years old) from Australia (48%, n = 58), United States (26%, n = 31), United Kingdom (12%, n = 14), New Zealand (9%, n = 11), the Netherlands (2%, n = 2), and not specified (4%, n = 5) representing a diverse injury profile, participated in the patient validation survey; 88% (n = 106) had lived experience of major injuries and 12% (n = 15) were caregivers. Fig. 6 shows the injury profile descriptions (e.g. injury type, mechanism and intent) that were scored by participants in the patient validation survey. It should be noted that participants could choose multiple descriptors. Seventy healthcare professionals, from Australia (33%, n = 23), New Zealand (16%, n = 11), United States (16%, n = 11), United Kingdom (10%, n = 7), Germany (4%, n = 3), India (4%, n = 3), Brazil (3%, n = 2), Canada (3%, n = 2), Ethiopia (1%, n = 1), Malaysia (1%, n = 1), the Netherlands (1%, n = 1), France (1%, n = 1), Norway (1%, n = 1), Philippines (1%, n = 1), Saudi Arabia (1%, n = 1), and South Africa (1%, n = 1), participated in the professional validation survey; 54 clinicians, 10 researchers, and 6 others (e.g. government professional, industry representative, etc.). 97% (n = 68) had civilian major injury experience and 9% (n = 6) had military experience. Experience with all injury types was represented. The baseline demographic characteristics of participants of the patient validation survey and the professionals open review survey are provided in the Supplementary material S4.

### Primary subset

The MJIWG was asked to vote on a PROM primary subset derived from the full set. The Patient-reported Health Status outcome measures endorsed for the primary set were pain (91%, n = 25), disability (96%, n = 27) and health-related Quality of Life (100%, n = 28). Measures endorsed in the Functioning domain were overall functioning (87%, n = 24), physical health (83%, n = 23), physical functioning (91%, n = 25), activities of daily living (87%, n = 24), and cognitive functioning (87%, n = 24). In the Psychological Well-Being/Mental Health domain, PTSD (87%, n = 24), depression (87%, n = 24), and anxiety (83%, n = 23) were endorsed. Mortality was also included as a clinical outcome (100%, n = 28). All case-mix variables were endorsed for the primary subset.

## Discussion

The ICHOM MJIWG has defined a set of PROMs and CROMs that can be used by healthcare professionals worldwide to support monitoring and evaluation of the treatment of patients with major injury regardless of their underlying health status, presence of specific conditions, and/or interventions. The set of outcome measures and variables was developed through a multi-methods process of consensus by an international working group with expertise relevant to outcomes assessment and care of patients with major injury. Importantly, patient input was critical, as was the use of published quantitative and qualitative data, and external validation. This led to the creation of a minimum set of outcomes, outcome measures, relevant case-mix variables, and time points overall representing the set of outcomes that matter to the patient. The MJIWG also defined a primary subset, which can be used as an implementation starting point. With both sets, outcomes can be compared between professionals and systems of care.

Major injuries are complex, and demand integrated multidisciplinary treatment. This set aims to integrate outcomes that matter to patients with major injury while recognising the unique needs of key patient groups (TBI, SCI and burns). Thus, the MJIWG also acknowledged the necessity for the development of specific, comprehensive ICHOM sets for TBI, SCI, and burns. The MJIWG did not reach consensus on inclusion of an extremity outcome measure as a special injury group, though the MJIWG did reach consensus on the measurement instrument (Short Musculoskeletal Function Assessment (SMFA)) as an appropriate tool should implementers wish to include one.

The major injury population is highly diverse with respect to the cause, nature, type, and severity of injury sustained, as well as the context in which the injury occurred. The MJIWG therefore recommended inclusion of these case-mix variables in the set to enable stratification of outcomes by injury circumstances and severity. Including the context of injury offers the advantage of extending the set's applicability beyond the civilian setting to collect both combat-related and non-combat scenarios of the military context. Outcome variables are expected to be more applicable in non-combat conditions, where data collection is generally more consistent and operational limitations are fewer. Applicability of the set to a military patient population is important to help military health systems facilitate benchmarking and improve quality of care.^24^

While comprehensive, not all outcomes, outcome measures or case-mix variables could be included in the set. For pragmatic reasons, literature searching was conducted in English. It is possible that potential outcomes or set items were not identified through the development process. However, the set was developed using a robust, established methodology which included wide geographical and diverse clinical and research expertise, patient input and external validation through professionals, patients and caregivers. The set was developed for use across the diverse major injury population and treatment settings and includes outcome measures that can be used worldwide at no cost. Moreover, the distribution of both the MJIWG and health professionals' open review survey was conducted across six continents, and three continents for the patient validation survey. A notable strength of the ICHOM methodology is the incorporation of validation surveys. However, it should be noted that the patient survey was constrained to samples from HIC populations, as ethical approval was exclusively granted in Australia, New Zealand, the Netherlands, the United Kingdom, and the United States. It was also recognised that the limited sample size and exclusive use of English language materials resulted in a lack of representativeness for the broader global population. Despite our best efforts to achieve an even broader distribution within the working group, making use of various networks, we were ultimately unable to achieve a proportional distribution of low-, middle- and high-income countries. A notable consideration is that the average participation rate of 68% (n = 19) across the Delphi surveys may limit the extent to which the achieved consensus fully represents the views of all expert panel members. However, it is noted that while MJIWG members may not have completed the survey, they remained involved in the panel discussions, thereby striving to provide maximum contribution and input.

In the context of future research and implementation trajectories, greater emphasis should be placed on low and middle-income countries in order to achieve a more equitable distribution. Nevertheless, the survey encompassed a diverse array of health professional roles, including trauma care clinicians, social care practitioners, policy advisers, researchers, advocacy/charity professionals, and commercial/industry representatives. The sample sizes for the patients (n = 121) and health professional survey (n = 70) were consistent with the sample sizes of similar ICHOM sets and exceeded the minimum sample size of 40 stipulated in the ICHOM methodology.

The study may also be limited by its focus on enhancing comprehensiveness through the use of multiple PROMs. Recommending administration of up to six PROMs per participant without empirical evidence on completion rates, patient fatigue, or clinical workflow feasibility risks undermining data quality and implementation, as highlighted by previous work on PROM burden and missing data.^25^ Future studies and implementations should evaluate the acceptability and real-world practicality of proposed PROM schedules. A further limitation concerns the selection of case-mix variables. This process is typically guided by four broad criteria—relevance, independence, practicality, and feasibility—yet these criteria are rarely accompanied by explicit operational definitions or weighting schemes. Instead, variables are usually chosen through consensus processes (e.g. modified Delphi panels), making the selection difficult to replicate and potentially influenced by the composition and preferences of expert groups rather than by standardised, objective evaluation. Finally, a scope that focuses on only including adults may miss an important group, namely children and adolescents. This group also faces major injury/trauma as a major cause of death and disability. The future development of a set for paediatric major injury is recommended, also based on the use of specific outcome measures for children.

The final set is available online via the ICHOM website: https://www.ichom.org/patient-centred-outcome-measure/major-injury/without cost. Healthcare institutions and/or healthcare systems, as well as individual healthcare professionals, can now implement the set. ICHOM has multiple examples of non-major injury implementation projects to assist and follow-up research is recommended to evaluate implementation of the set in major injury populations.^26^, ^27^, ^28^ Furthermore, the following examples come close to major injury implementation projects: (1) the Victorian State Trauma Registry is an exemplar of the routine implementation of PROMs in trauma care, systematically following up patients at 6, 12, and 24 months using measures such as EQ-5D to capture long-term recovery,^29^ and (2) in a similar manner, the New Zealand Trauma Registry has piloted PROMs collection in its national outcomes programme, thus facilitating consistent monitoring of patients' health status beyond survival. With this MJI core outcome set, those examples will even be more suited for example (international) comparison of data. It will be important to evaluate regional experiences and any challenges experienced in set implementation, including ensuring adaptability and transparency in the use of the set in research.^30^

The development and publication of the Patient-Centered Outcome Measures Set for Major Injury can be recognised as an important step towards understanding the health of and impact on major injury patients, using available and accessible measurement tools. The use of the set by healthcare professionals can contribute to the global reporting of outcomes data for performing comparisons in care delivered and patient outcomes achieved, and can also be used for shared learning, professional-patient dialogue, and creating value for the patient.

## Contributors

Conceptualisation: BG, HVDW, and WCDE. Study design: BG, HVDW, WCDE, UD, and YS. Data collection: HVDW, WCDE, UD, and YS. HVDW, WCDE, UD, and YS have access to and verify the underlying study data. Data analysis: BG, HVDW, WCDE, UD, and YS. Data interpretation: HVDW, WCDE, UD, YS, and BG. HVDW and WCDE wrote the original draft of the manuscript. Writing group: HVDW, WCDE, UD, YS, and BG. Reviewing and editing of the manuscript: BG, HVDW, WCDE, GJ, UD, and YS. All authors (including the MJI Working Group members) had access to the underlying data, reviewed and approved the final version of the manuscript. BG, HVDW, UD, and GJ had final responsibility for the decision to submit for publication.

## Data sharing statement

The Major Injury Set reference guide, data dictionary and flyer are available from ICHOM at no cost. The set can be accessed at https://www.ichom.org/patient-centred-outcome-measure/major-injury/. The reference guide contains detailed information on the recommended measurement tools, case-mix factors, and time points for data collection.

## Declaration of interests

HVDW, WCDE, UD, YS, GJ, SC, IC, DC, TC, CD, JHE, RH, KH, DM, JM, SN, WSP, MR, GS, MS, TVD, GZ: none declared; LMA: Royalties from Elsevier Australia for *Critical Care Nursing*; KC: charter member of a Loving Coalition Organisation; KM: Member Victorian Trauma System Advisory committee, ANZAST President; EM: Topic Adviser: National Institute for Health and Care Excellence UK, Member of Clinical Reference Group for Rehabilitation, Disability and Spinal Cord Injury NHS England; KL: declares being paid for attendance at meetings by ICHOM through the PCORI grant; KQ: Director Australia New Zealand Trauma Society, Member Health NZ–Trauma National Clinical Network; TSR: Research funding: Centers for Disease Control, National Institute of Minority Health & Disparities; TS: Speaker Korean Orthopaedic (&Trauma) Association, board member AO Foundation, and board member & president Fragility Fracture Network Global & Japan; MAS: Consultant and Grant recipient: Haemonetics and CSL Behring, DOD, and NIH, Consultant: Tricol, Velico Medical and Octapharma; HT: Grant recipient: National Institutes of Health/NCATS, and CDC; RVL: Employment by Dutch Ministry of Defence; BG: Grant recipient: NHMRC Australia, MRFF Australia, Transport Accident Commission, and Department of Health Victoria, and President of the Australian and New Zealand Trauma Society.
