# Supplementary material for: Development of a set of patient-centred outcome measures for patients with major injury: Delphi-based consensus recommendations from the International Consortium for Health Outcomes Measurement Major Injury Working Group
Source: eClinicalMedicine. 2025 Nov 3;90:103617. doi: 10.1016/j.eclinm.2025.103617 (PMC12766427; doi:10.1016/j.eclinm.2025.103617)
Supplement: Supplementary Materials [file mmc1.pdf]

Supplementary Materials
To the article: '*Development of a set of patient-centred outcome measures for patients with major injury: delphi-based consensus recommendations from the International Consortium for Health Outcomes Measurement Major Injury Working Group*'

Table of contents:

- Supplementary material 1 (S1) – Detailed (ICHOM) methodology
- Supplementary material 2 (S2) - Search strategies for the development of the set of patient-centred outcome measures for major injury
- Supplementary material 3 (S3) – Overview ethics committees and regulatory bodies providing ethics approval or exemption for the patient survey
- Supplementary material 4 (S4) – Baseline demographic characteristics of the Major Injury Working Group members and participants of the patient validation survey and professional open review survey
- Supplementary material 5 (S5) – Overview of psychometric properties of the PROMS selected by the Major Injury Working Group

Supplementary material 1 (S1) – Detailed (ICHOM) methodology

Introduction The modified-Delphi methodology used by the major injury working group (MJIWG) in this study follows the approach described by Mekonnen et al.[1], and other published ICHOM set.[2-5] All details and deviations are noted below.

The Major Injury Working Group The MJIWG included 28 professionals comprised clinical and research experts from numerous fields, ensuring a broad range of expertise; trauma surgery, trauma nursing, patient advocacy, public health, rehabilitation, and outcomes measurement research. They were recruited from 11 countries: Australia, Brazil, China, Japan, the Netherlands, New Zealand, Saudi Arabia, South Africa, Sweden, the United Kingdom, and the United States of America. In addition, the ICHOM project team (HVDW, WCDE, UD, YS) coordinated and facilitated the consensus-building process, and completed supporting research tasks, but did not vote with the Working Group on the recommendations to be made. The central project team included HVDW and WCDE as research fellows, UD as project manager, YS as research associate and BG as Working Group chair. The non-voting project team were based in the Netherlands, the UK, and Australia respectively.

Working Group members were identified through several avenues. Firstly, a rapid review was conducted by UD during the project initiation phase, to identify relevant service user organisations, measurement initiatives, professional bodies, and publications actively addressing questions relating to outcome measurement for major injury patients. Open recruitment calls inviting interested individuals to participate in the Working Group, Open Review process, or Set implementation were then disseminated through ICHOM's networks (via Newsletter and social media campaigns). The recruitment call was further disseminated by the funders of this initiative. Relevant organisations identified as part of the rapid review were contacted and the information shared. Individuals were identified through both routes. In the first instance, ICHOM identified a potential Working Group chair (BG), who was subsequently contacted and engaged. Next, a matrix of candidates was composed to facilitate the representation of diverse geographies, disciplines, and types of expertise (i.e., professional versus lived experience). A shortlist was created that would represent different matrix cells, and ICHOM subsequently invited shortlisted professionals to participate. There was a snowball sampling element to this process, where individuals or organizations recommended candidates for consideration by the ICHOM project team.[6] Patient representatives were invited based on their proactive solicitation of ICHOM in response to the open recruitment call. All participants were briefed on the project before agreeing to become part of the Working Group. All patient representatives who joined the Working Group participated throughout the entirety of the process and endorsed the final set. The other expert Working Group members participated on a voluntary basis.

The Consensus-Building Process Consensus was built through structured teleconferences, a modified-Delphi exercise, and iterative rounds of anonymous feedback surveys and voting, all informed by a systematic scoping review and other research inputs completed by the ICHOM project team.[7, 8] An open online consultation (henceforth called Open Review) gathered external feedback on the draft recommendations from researchers, practitioners, and service user representatives beyond the Working Group, towards the end of the consensus-building process.

Structured Teleconferences The Working Group convened for eight thematic structured teleconferences over a period of 12 months (May 2024 to May 2025). Professional Working Group members were required to participate in a minimum of 50% of the teleconferences and associated surveys to be considered full Working Group members. Patient representatives were expected to participate in the Delphi exercise, and strongly encouraged to also participate in all other stages of the process. On average, each teleconference was attended by 20 of the 28 Working Group members. Catch-up calls were offered to all members unable to make the full call. During each teleconference, the ICHOM project team (UD, YS) presented research findings (e.g., from the systematic scoping review or the appraisal of measurement instruments, see below), as well as anonymous voting results and comments from the modified-Delphi exercise and feedback surveys. Key decision points were suggested by the project team and discussed within the group. Participants also had the opportunity to raise additional issues or questions, or to come back to points raised at the end of each round. No formal decisions were made during teleconferences, and full minutes were shared with all Working Group members ahead of any voting.

Iterative Internal Feedback Surveys All key decisions about the Set were based on iterative voting via anonymous online surveys (via Qualtrics). These included decisions about the Set's scope, outcome domains, and recommendations in relation to measurement instruments, case-mix factors, and measurement time points. After each teleconference, a survey was circulated for members to vote on the key decision points identified during the call. Summaries of the research findings and discussion points from the corresponding conference call were provided for general reference, and to inform those who had been unable to attend. At least 80% of MJIWG

members had to vote in any given Working Group survey for the results to be considered valid; and every decision had to be endorsed by at least 70% of survey participants for consensus to be considered as reached.

The central project team (HVDW, WCDE, UD, YS) had a facilitating role and did not participate in the voting. Where required, initial voting results and free text comments from the survey were shared within the MJIWG during the subsequent call, to facilitate movement towards consensus in a subsequent round of voting. In some cases, the least favoured option from prior rounds was dropped, with the process made clear prior to voting. Areas of contention were openly discussed, and anonymous voting repeated until consensus was achieved. This structured process was used to reach consensus on the scope and recommendations of the Set. The MJIWG also voted to confirm their support on procedural aspects, such as the criteria for the selection of measurement instruments.

Modified-Delphi Exercise In order to determine the outcome domains for inclusion, a modified three-round Delphi process was run following the second teleconference. As in all MJIWG surveys, 80% participation was required in each round. In the first round, each voting MJIWG member ranked each potential outcome domain on a scale from 1 to 9 based on a number of criteria, to indicate whether they thought the domain should be included in the Set. This was in line with common practice in Delphi surveys, where 9-point rating scales are frequently used.[(9)] Domains ranked between 7 and 9 by 80% of the MJIWG were included after round one. Those domains that were ranked between 1 and 3 by 80% of the MJIWG were excluded. For all outcome domains that fell in between (4 through 6), were considered inconclusive and written comments provided by MJIWG members in the Delphi survey were shared. A second round of voting with the same process was then completed. After the third teleconference, all remaining ambiguous outcome domains were discussed during a Working Group call and then subject to a binary Yes/No inclusion vote, with 70% consensus required for inclusion. The rationale for the 70% threshold in the final round is twofold. Firstly, it is for consistency with other research on consensus methodologies, where many Delphi processes in the health outcomes literature use thresholds ranging from 60-80% for consensus. A 70% threshold is accepted as being sufficient to indicate group agreement while allowing minority perspectives to be acknowledged. Secondly, the threshold is lowered to strike a balance between rigor and feasibility. The final Delphi survey involves decisions on a smaller set of outcomes that have gone through several rounds of review and discussion, and at this stage, using a 70% threshold ensures that items with broad (though not absolute) support are not excluded, particularly because multidisciplinary and international views are being represented.

As described above, decisions about other aspects of the Set (i.e., scope, measurement instruments, case-mix factors, and time points) were reached using simplified voting techniques and rating scales, including binary response options, and choices between multiple alternative options.

References

1. Mekonnen T, Staniford L, Connell S, Das-Gupta Z, DeSilva U, Saoud Y, et al. Development of a core patient-centred outcome set for adults living with obesity: a modified delphi-based international consensus. EClinicalMedicine. 2025;87:103422.
2. Wouters RM, Jobi-Odeneye AO, de la Torre A, Joseph A, Hand I, Wrist Working G, et al. A Standard Set for Outcome Measurement in Patients With Hand and Wrist Conditions: Consensus by the International Consortium for Health Outcomes Measurement Hand and Wrist Working Group. J Hand Surg Am. 2021.
3. Mulraney M, de Silva U, Joseph A, Sousa Fialho MDL, Dutia I, Munro N, et al. International Consensus on Standard Outcome Measures for Neurodevelopmental Disorders: A Consensus Statement. JAMA Netw Open. 2024;7(6):e2416760.
4. Krause KR, Chung S, Adewuya AO, Albano AM, Babins-Wagner R, Birkinshaw L, et al. International consensus on a standard set of outcome measures for child and youth anxiety, depression, obsessive-compulsive disorder, and post-traumatic stress disorder. Lancet Psychiatry. 2021;8(1):76–86.
5. Seligman WH, Das-Gupta Z, Jobi-Odeneye AO, Arbelo E, Banerjee A, Bollmann A, et al. Development of an international standard set of outcome measures for patients with atrial fibrillation: a report of the International Consortium for Health Outcomes Measurement (ICHOM) atrial fibrillation working group. Eur Heart J. 2020;41(10):1132–40.
6. Biernacki P, Waldorf D. Snowball Sampling: Problems and Techniques of Chain Referral Sampling. Sociological Methods & Research. 1981;10(2):141–63.
7. Fitch K. The Rand/UCLA appropriateness method user's manual. Santa Monica: Rand; 2001.
8. Boulkedid R, Abdoul H, Loustau M, Sibony O, Alberti C. Using and reporting the Delphi method for selecting healthcare quality indicators: a systematic review. PLoS One. 2011;6(6):e20476.
9. Fleiss JL. Statistical methods for rates and proportions. 2d ed. New York: Wiley; 1981. xviii, 321 p. p.

Supplementary material 2 (S2) - Search strategies for the development of the set of patient-centred outcome measures for major injury

Pubmed

**Database:**	Pubmed	13-06-2024	
**General search**	Search words:	Number of records:	
#1	"WOUNDS AND INJURIES" [MeSH]Su NOT "Asphyxia" [MeSH] NOT "Barotrauma" [MeSH] NOT "Birth injuries" [MeSH] NOT "Bites and Stings" [MeSH] NOT "Drowning" [MeSH] NOT "Heat Stress Disorders" [MeSH] NOT "Radiation injuries" [MeSH] NOT "Tooth injuries" [MeSH]	889.621	
#2	"ACUTE CARE SURGERY"[MeSH]	3.923	
#3	("ORTHOPEDICS" [MeSH] OR ("orthop*edics"[Tiab] adj3 ("trauma*" OR "polytrauma*" OR "multiple trauma*" OR (("serious*" OR "severe*" OR "major" OR "life threaten*") adj3 ("accident*" OR "injur*" OR "fall")))))	24.750	
#4	"BRAIN INJURIES, TRAUMATIC" [MeSH]	26.488	
#5	1 or 2 or 3 or 4	912.116	WOUNDS AND INJURIES [MeSH] NOT "Asphyxia" [MeSH] NOT "Barotrauma" [MeSH] NOT "Birth injuries" [MeSH] NOT "Bites and Stings" [MeSH] NOT "Drowning" [MeSH] NOT "Heat Stress Disorders" [MeSH] NOT "Radiation injuries" [MeSH] NOT "Tooth injuries" [MeSH] OR "ACUTE CARE SURGERY"[MeSH] OR ("ORTHOPEDICS" [MeSH] OR ("orthop*edics"[Tiab] adj3 ("trauma*" OR "polytrauma*" OR "multiple trauma*" OR (("serious*" OR "severe*" OR "major" OR "life threaten*") adj3 ("accident*" OR "injur*" OR "fall"))))) OR "BRAIN INJURIES, TRAUMATIC" [MeSH]
#6	"PSYCHOLOGICAL TRAUMA" [MeSH]	2.077	
#7	5 not 6	912.060	(WOUNDS AND INJURIES [MeSH] NOT "Asphyxia" [MeSH] NOT "Barotrauma" [MeSH] NOT "Birth injuries" [MeSH] NOT "Bites and Stings" [MeSH] NOT "Drowning" [MeSH] NOT "Heat Stress Disorders" [MeSH] NOT "Radiation injuries" [MeSH] NOT "Tooth injuries" [MeSH] OR "ACUTE CARE SURGERY"[MeSH] OR ("ORTHOPEDICS" [MeSH] OR ("orthop*edics"[Tiab] adj3 ("trauma*" OR "polytrauma*" OR "multiple trauma*" OR (("serious*" OR "severe*" OR "major" OR "life threaten*") adj3 ("accident*" OR "injur*" OR "fall"))))) OR "BRAIN INJURIES, TRAUMATIC" [MeSH]) NOT "PSYCHOLOGICAL TRAUMA" [MeSH]
#8	"OUTCOME ASSESSMENT, HEALTH CARE" [MeSH]	1.385.310	
#9	"PATIENT OUTCOME ASSESSMENT" [MeSH]	24.326	
#10	"QUALITY OF LIFE" [MeSH]	289.601	
#11	("patient reported outcome*" OR "patient-reported outcome*" OR "patient-related outcome*" OR "patient related outcome*")	47.831	
#12	(hrql OR hrqol OR qol OR ql OR "quality of life")	593.895	
#13	(index OR indices OR instrument* OR measure* OR questionnaire* OR profile* OR scale* OR score* OR status OR survey*)	12.390.046	

#14	12 and 13	390.212	((hrql OR hrqol OR qol OR ql OR "quality of life") AND (index OR indices OR instrument* OR measure* OR questionnaire* OR profile* OR scale* OR score* OR status OR survey*)
#15	8 or 9 or 10 or 11 or 14	1.783.855	"OUTCOME ASSESSMENT, HEALTH CARE" [MeSH] OR "PATIENT OUTCOME ASSESSMENT" [MeSH] OR "QUALITY OF LIFE" [MeSH] OR ("patient reported outcome*" OR "patient-reported outcome*" OR "patient-related outcome*" OR "patient related outcome*") OR ((hrql OR hrqol OR qol OR ql OR "quality of life") AND (index OR indices OR instrument* OR measure* OR questionnaire* OR profile* OR scale* OR score* OR status OR survey*))
#16	7 and 15	108.441	((WOUNDS AND INJURIES [MeSH] NOT "Asphyxia" [MeSH] NOT "Barotrauma" [MeSH] NOT "Birth injuries" [MeSH] NOT "Bites and Stings" [MeSH] NOT "Drowning" [MeSH] NOT "Heat Stress Disorders" [MeSH] NOT "Radiation injuries" [MeSH] NOT "Tooth injuries" [MeSH] OR "ACUTE CARE SURGERY"[MeSH] OR ("ORTHOPEDICS" [MeSH] OR ("orthop*edics"[Tiab] adj3 ("trauma*" OR "polytrauma*" OR "multiple trauma*" OR (("serious*" OR "severe*" OR "major" OR "life threaten*") adj3 ("accident*" OR "injur*" OR "fall"))))) OR "BRAIN INJURIES, TRAUMATIC" [MeSH]) NOT "PSYCHOLOGICAL TRAUMA" [MeSH]) AND ("OUTCOME ASSESSMENT, HEALTH CARE" [MeSH] OR "PATIENT OUTCOME ASSESSMENT" [MeSH] OR "QUALITY OF LIFE" [MeSH] OR ("patient reported outcome*" OR "patient-reported outcome*" OR "patient-related outcome*" OR "patient related outcome*") OR ((hrql OR hrqol OR qol OR ql OR "quality of life") AND (index OR indices OR instrument* OR measure* OR questionnaire* OR profile* OR scale* OR score* OR status OR survey*)))
#17	"ADULT" [MeSH]	8.092.036	
#18	"AGED" [MeSH] OR "geriatric*" [Tiab] OR "elderl*" [Tiab] OR "older adult*" [Tiab] OR "older patient*" [Tiab] OR "older man" [Tiab] OR "older men" [Tiab] OR "older male" [Tiab] OR "older woman" [Tiab] OR "older women" [Tiab] OR "older female" [Tiab] OR "older person*" [Tiab] OR "frail*" [Tiab] OR "senior*" [Tiab]	3.732.989	
#19	17 or 18	8.271.351	"ADULT" [MeSH] OR "AGED" [MeSH] OR "geriatric*" [Tiab] OR "elderl*" [Tiab] OR "older adult*" [Tiab] OR "older patient*" [Tiab] OR "older man" [Tiab] OR "older men" [Tiab] OR "older male" [Tiab] OR "older woman" [Tiab] OR "older women" [Tiab] OR "older female" [Tiab] OR "older person*" [Tiab] OR "frail*" [Tiab] OR "senior*" [Tiab]
#20	"PEDIATRICS" [MeSH]	63.640	
#21	19 not 20	8.264.371	("ADULT" [MeSH] OR "AGED" [MeSH] OR "geriatric*" [Tiab] OR "elderl*" [Tiab] OR "older adult*" [Tiab] OR "older patient*" [Tiab] OR "older man" [Tiab] OR "older men" [Tiab] OR "older male" [Tiab] OR "older woman" [Tiab] OR "older women" [Tiab] OR "older female" [Tiab] OR "older person*" [Tiab] OR "frail*" [Tiab] OR "senior*" [Tiab]) NOT "PEDIATRICS" [MeSH]
#22	16 and 21	71.900	((WOUNDS AND INJURIES [MeSH] NOT "Asphyxia" [MeSH] NOT "Barotrauma" [MeSH] NOT "Birth injuries" [MeSH] NOT "Bites and Stings" [MeSH] NOT "Drowning" [MeSH] NOT "Heat Stress Disorders" [MeSH] NOT "Radiation injuries" [MeSH] NOT "Tooth injuries" [MeSH] OR "ACUTE CARE SURGERY"[MeSH] OR ("ORTHOPEDICS" [MeSH] OR ("orthop*edics"[Tiab] adj3 ("trauma*" OR "polytrauma*" OR "multiple trauma*" OR (("serious*" OR "severe*" OR "major" OR "life threaten*") adj3

			(“accident*” OR “injur*” OR “fall”))))) OR “BRAIN INJURIES, TRAUMATIC” [MeSH]) NOT “PSYCHOLOGICAL TRAUMA” [MeSH]) AND (“OUTCOME ASSESSMENT, HEALTH CARE” [MeSH] OR “PATIENT OUTCOME ASSESSMENT” [MeSH] OR “QUALITY OF LIFE” [MeSH] OR (“patient reported outcome*” OR “patient-reported outcome*” OR “patient-related outcome*” OR “patient related outcome*”) OR ((hrql OR hrqol OR qol OR ql OR “quality of life”) AND (index OR indices OR instrument* OR measure* OR questionnaire* OR profile* OR scale* OR score* OR status OR survey*))) AND ((“ADULT” [MeSH] OR “AGED” [MeSH] OR “geriatric*” [Tiab] OR “elderl*” [Tiab] OR “older adult*” [Tiab] OR “older patient*” [Tiab] OR “older man” [Tiab] OR “older men” [Tiab] OR “older male” [Tiab] OR “older woman” [Tiab] OR “older women” [Tiab] OR “older female” [Tiab] OR “older person*” [Tiab] OR “frail*” [Tiab] OR “senior*” [Tiab]) NOT “PEDIATRICS” [MeSH])
#23	“CLINICAL TRIAL” [publication type]	996.068	
#24	“SYSTEMATIC” [sb]	311.686	
#25	(“RCT” [Tiab] OR “controlled trial*” [Tiab] OR “control trial*” [Tiab] OR “clinical trial*” [Tiab] OR “clinicaltrial*” [Tiab] OR “open label trial*” [Tiab] OR “open trial*” [Tiab] OR “randomi*ed trial*” [Tiab])	955.016	
#26	23 or 24 or 25	1.862.986	“CLINICAL TRIAL” [publication type] OR “SYSTEMATIC” [sb] OR (“RCT” [Tiab] OR “controlled trial*” [Tiab] OR “control trial*” [Tiab] OR “clinical trial*” [Tiab] OR “clinicaltrial*” [Tiab] OR “open label trial*” [Tiab] OR “open trial*” [Tiab] OR “randomi*ed trial*” [Tiab])
#27	“OBSERVATIONAL STUDY” [publication type]	156.912	
#28	“REVIEW” [publication type]	89.147	
#29	“QUALITATIVE RESEARCH” [MeSH]	112.226	
#30	“COHORT STUDIES” [MeSH]	2.616.911	
#31	(“case series”[Tiab] OR ((“naturalistic”[Tiab] OR “case-control”[Tiab]) adj1 (“stud*”[Tiab] OR “design*”[Tiab] OR “trial*”[Tiab])) OR (“qualitative”[Tiab] adj1 (“stud*”[Tiab] OR “research”[Tiab] OR “design*”[Tiab] OR “interview*”[Tiab])))	3.339.512	
#32	27 or 28 or 29 or 30 or 31	6.076.965	“OBSERVATIONAL STUDY” [publication type] OR “QUALITATIVE RESEARCH” [MeSH] OR “COHORT STUDIES” [MeSH] OR (“case series”[Tiab] OR ((“naturalistic”[Tiab] OR “case-control”[Tiab]) adj1 (“stud*”[Tiab] OR “design*”[Tiab] OR “trial*”[Tiab])) OR (“qualitative”[Tiab] adj1 (“stud*”[Tiab] OR “research”[Tiab] OR “design*”[Tiab] OR “interview*”[Tiab]))) OR “REVIEW” [publication type]
#33	26 or 32	7.252.185	“CLINICAL TRIAL” [publication type] OR “SYSTEMATIC” [sb] OR (“RCT” [Tiab] OR “controlled trial*” [Tiab] OR “control trial*” [Tiab] OR “clinical trial*” [Tiab] OR “clinicaltrial*” [Tiab] OR “open label trial*” [Tiab] OR “open trial*” [Tiab] OR “randomi*ed trial*” [Tiab]) OR “OBSERVATIONAL STUDY” [publication type] OR “QUALITATIVE RESEARCH” [MeSH] OR “COHORT STUDIES” [MeSH] OR (“case series”[Tiab] OR ((“naturalistic”[Tiab] OR “case-control”[Tiab]) adj1 (“stud*”[Tiab] OR “design*”[Tiab] OR “trial*”[Tiab])) OR (“qualitative”[Tiab] adj1 (“stud*”[Tiab] OR “research”[Tiab] OR “design*"[Tiab] OR "interview*"[Tiab]))) OR "REVIEW" [publication type]

#34	22 and 33	48.631	((WOUNDS AND INJURIES [MeSH] NOT "Asphyxia" [MeSH] NOT "Barotrauma" [MeSH] NOT "Birth injuries" [MeSH] NOT "Bites and Stings" [MeSH] NOT "Drowning" [MeSH] NOT "Heat Stress Disorders" [MeSH] NOT "Radiation injuries" [MeSH] NOT "Tooth injuries" [MeSH] OR "ACUTE CARE SURGERY"[MeSH] OR ("ORTHOPEDICS" [MeSH] OR ("orthop*edics"[Tiab] adj3 ("trauma*" OR "polytrauma*" OR "multiple trauma*" OR (("serious*" OR "severe*" OR "major" OR "life threaten*") adj3 ("accident*" OR "injur*" OR "fall"))))) OR "BRAIN INJURIES, TRAUMATIC" [MeSH]) NOT "PSYCHOLOGICAL TRAUMA" [MeSH]) AND ("OUTCOME ASSESSMENT, HEALTH CARE" [MeSH] OR "PATIENT OUTCOME ASSESSMENT" [MeSH] OR "QUALITY OF LIFE" [MeSH] OR ("patient reported outcome*" OR "patient-reported outcome*" OR "patient-related outcome*" OR "patient related outcome*") OR ((hrql OR hrqol OR qol OR ql OR "quality of life") AND (index OR indices OR instrument* OR measure* OR questionnaire* OR profile* OR scale* OR score* OR status OR survey*))) AND (("ADULT" [MeSH] OR "AGED" [MeSH] OR "geriatric*" [Tiab] OR "elderl*" [Tiab] OR "older adult*" [Tiab] OR "older patient*" [Tiab] OR "older man" [Tiab] OR "older men" [Tiab] OR "older male" [Tiab] OR "older woman" [Tiab] OR "older women" [Tiab] OR "older female" [Tiab] OR "older person*" [Tiab] OR "frail*" [Tiab] OR "senior*" [Tiab]) NOT "PEDIATRICS" [MeSH]) AND ("CLINICAL TRIAL" [publication type] OR "SYSTEMATIC" [sb] OR ("RCT" [Tiab] OR "controlled trial*" [Tiab] OR "control trial*" [Tiab] OR "clinical trial*" [Tiab] OR "clinicaltrial*" [Tiab] OR "open label trial*" [Tiab] OR "open trial*" [Tiab] OR "randomi*ed trial*" [Tiab]) OR "OBSERVATIONAL STUDY" [publication type] OR "QUALITATIVE RESEARCH" [MeSH] OR "COHORT STUDIES" [MeSH] OR ("case series"[Tiab] OR (("naturalistic"[Tiab] OR "case-control"[Tiab]) adj1 ("stud*"[Tiab] OR "design*"[Tiab] OR "trial*"[Tiab])) OR ("qualitative"[Tiab] adj1 ("stud*"[Tiab] OR "research"[Tiab] OR "design*"[Tiab] OR "interview*"[Tiab]))) OR "REVIEW" [publication type])
#35	2005:2023 [dp]		
#36	34 and 35	39.221	((WOUNDS AND INJURIES [MeSH] NOT "Asphyxia" [MeSH] NOT "Barotrauma" [MeSH] NOT "Birth injuries" [MeSH] NOT "Bites and Stings" [MeSH] NOT "Drowning" [MeSH] NOT "Heat Stress Disorders" [MeSH] NOT "Radiation injuries" [MeSH] NOT "Tooth injuries" [MeSH] OR "ACUTE CARE SURGERY"[MeSH] OR ("ORTHOPEDICS" [MeSH] OR ("orthop*edics"[Tiab] adj3 ("trauma*" OR "polytrauma*" OR "multiple trauma*" OR (("serious*" OR "severe*" OR "major" OR "life threaten*") adj3 ("accident*" OR "injur*" OR "fall"))))) OR "BRAIN INJURIES, TRAUMATIC" [MeSH]) NOT "PSYCHOLOGICAL TRAUMA" [MeSH]) AND ("OUTCOME ASSESSMENT, HEALTH CARE" [MeSH] OR "PATIENT OUTCOME ASSESSMENT" [MeSH] OR "QUALITY OF LIFE" [MeSH] OR ("patient reported outcome*" OR "patient-reported outcome*" OR "patient-related outcome*" OR "patient related outcome*") OR ((hrql OR hrqol OR qol OR ql OR "quality of life") AND (index OR indices OR instrument* OR measure* OR questionnaire* OR profile* OR scale* OR score* OR status OR survey*))) AND (("ADULT" [MeSH] OR "AGED" [MeSH] OR "geriatric*" [Tiab] OR "elderl*" [Tiab] OR "older adult*" [Tiab] OR "older patient*" [Tiab] OR "older man" [Tiab] OR "older men" [Tiab] OR "older male" [Tiab] OR "older woman" [Tiab] OR "older women" [Tiab] OR "older female" [Tiab] OR "older person*" [Tiab] OR "frail*" [Tiab] OR "senior*" [Tiab]) NOT "PEDIATRICS" [MeSH]) AND ("CLINICAL TRIAL" [publication type] OR "SYSTEMATIC" [sb] OR

			("RCT" [Tiab] OR "controlled trial*" [Tiab] OR "control trial*" [Tiab] OR "clinical trial*" [Tiab] OR "clinicaltrial*" [Tiab] OR "open label trial*" [Tiab] OR "open trial*" [Tiab] OR "randomi*ed trial*" [Tiab]) OR "OBSERVATIONAL STUDY" [publication type] OR "QUALITATIVE RESEARCH" [MeSH] OR "COHORT STUDIES" [MeSH] OR ("case series"[Tiab] OR (("naturalistic"[Tiab] OR "case-control"[Tiab]) adj1 ("stud*"[Tiab] OR "design*"[Tiab] OR "trial*"[Tiab])) OR ("qualitative"[Tiab] adj1 ("stud*"[Tiab] OR "research"[Tiab] OR "design*"[Tiab] OR "interview*"[Tiab]))) OR "REVIEW" [publication type]) AND 2005:2023 [dp]
	English [la]		
	36 and 37	36.508	((WOUNDS AND INJURIES [MeSH] NOT "Asphyxia" [MeSH] NOT "Barotrauma" [MeSH] NOT "Birth injuries" [MeSH] NOT "Bites and Stings" [MeSH] NOT "Drowning" [MeSH] NOT "Heat Stress Disorders" [MeSH] NOT "Radiation injuries" [MeSH] NOT "Tooth injuries" [MeSH] OR "ACUTE CARE SURGERY"[MeSH] OR ("ORTHOPEDICS" [MeSH] OR ("orthop*edics"[Tiab] adj3 ("trauma*" OR "polytrauma*" OR "multiple trauma*" OR (("serious*" OR "severe*" OR "major" OR "life threaten*") adj3 ("accident*" OR "injur*" OR "fall"))))) OR "BRAIN INJURIES, TRAUMATIC" [MeSH]) NOT "PSYCHOLOGICAL TRAUMA" [MeSH]) AND ("OUTCOME ASSESSMENT, HEALTH CARE" [MeSH] OR "PATIENT OUTCOME ASSESSMENT" [MeSH] OR "QUALITY OF LIFE" [MeSH] OR ("patient reported outcome*" OR "patient-reported outcome*" OR "patient-related outcome*" OR "patient related outcome*") OR ((hrql OR hrqol OR qol OR ql OR "quality of life") AND (index OR indices OR instrument* OR measure* OR questionnaire* OR profile* OR scale* OR score* OR status OR survey*))) AND (("ADULT" [MeSH] OR "AGED" [MeSH] OR "geriatric*" [Tiab] OR "elderl*" [Tiab] OR "older adult*" [Tiab] OR "older patient*" [Tiab] OR "older man" [Tiab] OR "older men" [Tiab] OR "older male" [Tiab] OR "older woman" [Tiab] OR "older women" [Tiab] OR "older female" [Tiab] OR "older person*" [Tiab] OR "frail*" [Tiab] OR "senior*" [Tiab]) NOT "PEDIATRICS" [MeSH]) AND ("CLINICAL TRIAL" [publication type] OR "SYSTEMATIC" [sb] OR ("RCT" [Tiab] OR "controlled trial*" [Tiab] OR "control trial*" [Tiab] OR "clinical trial*" [Tiab] OR "clinicaltrial*" [Tiab] OR "open label trial*" [Tiab] OR "open trial*" [Tiab] OR "randomi*ed trial*" [Tiab]) OR "OBSERVATIONAL STUDY" [publication type] OR "QUALITATIVE RESEARCH" [MeSH] OR "COHORT STUDIES" [MeSH] OR ("case series"[Tiab] OR (("naturalistic"[Tiab] OR "case-control"[Tiab]) adj1 ("stud*"[Tiab] OR "design*"[Tiab] OR "trial*"[Tiab])) OR ("qualitative"[Tiab] adj1 ("stud*"[Tiab] OR "research"[Tiab] OR "design*"[Tiab] OR "interview*"[Tiab]))) OR "REVIEW" [publication type]) AND 2005:2023 [dp] AND English [la]

((WOUNDS AND INJURIES [MeSH] NOT "Asphyxia" [MeSH] NOT "Barotrauma" [MeSH] NOT "Birth injuries" [MeSH] NOT "Bites and Stings" [MeSH] NOT "Drowning" [MeSH] NOT "Heat Stress Disorders" [MeSH] NOT "Radiation injuries" [MeSH] NOT "Tooth injuries" [MeSH] OR "ACUTE CARE SURGERY"[MeSH] OR ("ORTHOPEDICS" [MeSH] OR ("orthop*edics"[Tiab] adj3 ("trauma*" OR "polytrauma*" OR "multiple trauma*" OR (("serious*" OR "severe*" OR "major" OR "life threaten*") adj3 ("accident*" OR "injur*" OR "fall"))))) OR "BRAIN INJURIES, TRAUMATIC" [MeSH]) NOT "PSYCHOLOGICAL TRAUMA" [MeSH]) AND ("OUTCOME ASSESSMENT, HEALTH CARE" [MeSH] OR "PATIENT OUTCOME ASSESSMENT" [MeSH] OR "QUALITY OF LIFE" [MeSH] OR ("patient reported outcome*" OR "patient-reported outcome*" OR "patient-related outcome*" OR "patient related outcome*") OR ((hrql OR hrqol OR qol OR ql OR "quality of life") AND (index OR indices OR instrument* OR measure* OR questionnaire* OR profile* OR scale* OR score* OR status OR survey*))) AND (("ADULT" [MeSH] OR "AGED" [MeSH] OR "geriatric*" [Tiab] OR "elderl*" [Tiab] OR "older adult*" [Tiab] OR "older patient*" [Tiab] OR "older man" [Tiab] OR "older men" [Tiab] OR "older male" [Tiab] OR "older woman" [Tiab] OR

"older women" [Tiab] OR "older female" [Tiab] OR "older person*" [Tiab] OR "frail*" [Tiab] OR "senior*" [Tiab]) NOT "PEDIATRICS" [MeSH]) AND ("CLINICAL TRIAL" [publication type] OR "SYSTEMATIC" [sb] OR ("RCT" [Tiab] OR "controlled trial*" [Tiab] OR "control trial*" [Tiab] OR "clinical trial*" [Tiab] OR "clinicaltrial*" [Tiab] OR "open label trial*" [Tiab] OR "open trial*" [Tiab] OR "randomi*ed trial*" [Tiab]) OR "OBSERVATIONAL STUDY" [publication type] OR "QUALITATIVE RESEARCH" [MeSH] OR "COHORT STUDIES" [MeSH] OR ("case series"[Tiab] OR (("naturalistic"[Tiab] OR "case-control"[Tiab]) adj1 ("stud*"[Tiab] OR "design*"[Tiab] OR "trial*"[Tiab])) OR ("qualitative"[Tiab] adj1 ("stud*"[Tiab] OR "research"[Tiab] OR "design*"[Tiab] OR "interview*"[Tiab]))) OR "REVIEW" [publication type]) AND 2005:2023 [dp] AND English [la]

Embase

acute care surgery'/ OR 'traumatic brain injury'/ OR 'injury' or exp 'asphyxia' or exp 'barotrauma'/ or exp 'birth injury'/ or exp 'bite'/ or exp 'sting'/ or exp 'drowning'/ or exp 'heat stress disorder'/ or exp 'radiation injury'/ or exp 'tooth injury'/ or exp 'poisoning'/ or exp 'battered child syndrom'/ or exp 'chemical injury'/ or exp 'prenatal injury'/ OR 'orthop*edic'/ or exp 'psychological trauma'/ AND ('outcome assessment'/ OR 'patient outcome assessment'/ OR 'quality of life'/ OR 'patient reported outcome*' OR 'patient-reported outcome*' OR 'patient-related outcome*' OR 'patient related outcome*') AND ('adult'/ OR 'aged'/ OR geriatric* OR elderl* OR 'older adult*' OR 'older patient*' OR 'older man' OR 'older men' OR 'older male' OR 'older woman' OR 'older women' OR 'older female' OR 'older person*' OR frail* OR senior* OR exp 'pediatrics'/) AND ('clinical trial'/ OR 'systematic review'/ OR 'rct' OR 'controlled trial*' OR 'control trial*' OR 'clinical trial*' OR 'clinicaltrial*' OR 'open label trial*' OR 'open trial*' OR 'randomized trial*' OR 'randomised trial*' OR 'observational study' OR 'qualitative research' OR 'cohort analysis' OR 'case series' OR 'review')

PsycINFO

((DE "Wounds" OR DE "Injuries") NOT (DE "Asphyxia" OR DE "Barotrauma" OR DE "Birth Injuries" OR DE "Bites and Stings" OR DE "Drowning" OR DE "Heat Stress" OR DE "Radiation Injuries" OR DE "Tooth Injuries") OR DE "Acute Care" OR (DE "Orthopedic Surgery" OR (TI (orthopedics OR orthopaedics) AND (trauma* OR polytrauma* OR "multiple trauma*" OR (serious* OR severe* OR major OR "life threaten*") AND (accident* OR injur* OR fall)))) OR DE "Traumatic Brain Injury") NOT DE "Psychological Trauma") AND (DE "Outcome Assessment" OR DE "Patient Outcome" OR DE "Quality of Life" OR (AB "patient reported outcome*" OR AB "patient-reported outcome*" OR AB "patient-related outcome*" OR AB "patient related outcome*") OR (AB (hrql OR hrqol OR qol OR ql OR "quality of life") AND (index OR indices OR instrument* OR measure* OR questionnaire* OR profile* OR scale* OR score* OR status OR survey*))) AND (DE "Adulthood" OR DE "Aging" OR (AB geriatric* OR elderl* OR "older adult*" OR "older patient*" OR "older man" OR "older men" OR "older male" OR "older woman" OR "older women" OR "older female" OR "older person*" OR frail* OR senior*)) NOT DE "Pediatrics") AND (DE "Clinical Trials" OR DE "Systematic Review" OR (AB "RCT" OR AB "controlled trial*" OR AB "control trial*" OR AB "clinical trial*" OR AB "clinicaltrial*" OR AB "open label trial*" OR AB "open trial*" OR AB "randomized trial" OR AB "randomised trial") OR DE "Observational Study" OR DE "Qualitative Research" OR DE "Cohort Studies")

PROM extraction

#1.1 Patient-reported health status
(“patient reported health status”[tw] OR “symptom severity”[tw] OR “pain”[tw] OR “disability”[tw] OR “HRQol”[tw] OR “EQ-5D-5L”[tw] OR “EuroQol-5D”[tw] OR “Munich Shoulder Questionnaire”[tw] OR “Shoulder Pain and Disability Index”[tw] OR “SEIQoL-DW”[tw] OR “AQoL”[tw] OR “Visual Analogue Scale”[tw] OR “SF-12”[tw] OR “Satisfaction with Life Scale”[tw] OR “WHOQOL-Bref”[tw] OR “SF-36”[tw] OR “pain intensity”[tw] OR “S-LANSS”[tw] OR “Brief Pain Inventory”[tw] OR “QoL-BDS”[tw] OR “NPRS”[tw] OR “WHO-QOL”[tw] OR “DASH”[tw] OR “PRWE”[tw] OR “Quality of Life After Brain Injury”[tw] OR “FOAS”[tw] OR “PROMIS-29”[tw] OR “HIT-6”[tw] OR “FJS-12”[tw])

#1.2 Psychological wellbeing/mental health
(“mental health”[MeSH] OR (“mental”[Tiab] adj3 “health”[Tiab]) OR “mental function”[Tiab] OR “psychological function*”[Tiab] OR “psychological consequenc*”[Tiab] OR “Psychological wellbeing”[Tiab] OR “psychological health” OR “Substance-related disorders”[MeSH] OR “substance use”[Tiab] OR "substance abuse"[Tiab] OR "substance use disorder"[Tiab] OR "substance misuse"[Tiab] OR “drug use”[Tiab] OR “alcohol abuse”[Tiab] OR “alcoholism”[Tiab] OR “dependency”[Tiab] OR “addiction”[Tiab] OR (“drug”[Tiab] adj3

“abuse”[Tiab]) OR “PTSD”[Tiab] OR “posttraumatic stress”[Tiab] or “posttraumatic stress disorder*”[Tiab] or “post-traumatic stress disorder*”[Tiab])

#1.3 Functioning
(“overall functioning”[Tiab] OR “functional status”[Tiab] OR “physical health”[Tiab] OR (“physical”[Tiab] adj3 “health”[Tiab]) OR “global functioning”[Tiab] OR (“physical”[Tiab] adj3 ”functioning”[Tiab]) OR (“social”[Tiab] adj3 “functioning”[Tiab]) OR “activities of dialy living”[MeSH] OR “daily activities”[Tiab] OR “activities of daily life”[Tiab] OR “activities of daily living”[Tiab] OR “ADL”[Tiab] OR “ADLs”[Tiab] OR “basic ADL”[Tiab] OR “Instrumental ADL”[Tiab] OR “occupational functioning”[Tiab] OR “occupational function*”[Tiab] OR (“occupational”[Tiab] adj3 “function*”[Tiab]) OR “occupational health”[MeSH] OR “work health”[Tiab] OR “work status”[Tiab] OR (“work”[Tiab] adj3 “function*”[Tiab]) OR “occupational status”[Tiab] OR (“occupation*”[Tiab] adj3 “function*”[Tiab]) OR “cognitive function*”[Tiab] OR “cognitive health”[Tiab] OR (“cognitive*”[Tiab] adj3 “function*”[Tiab]))

AND #2
((WOUNDS AND INJURIES [MeSH] NOT "Asphyxia" [MeSH] NOT "Barotrauma" [MeSH] NOT "Birth injuries" [MeSH] NOT "Bites and Stings" [MeSH] NOT "Drowning" [MeSH] NOT "Heat Stress Disorders" [MeSH] NOT "Radiation injuries" [MeSH] NOT "Tooth injuries" [MeSH] OR "ACUTE CARE SURGERY"[MeSH] OR ("ORTHOPEDICS" [MeSH] OR ("orthop*edics"[Tiab] adj3 ("trauma*" OR "polytrauma*" OR "multiple trauma*" OR (("serious*" OR "severe*" OR "major" OR "life threaten*") adj3 ("accident*" OR "injur*" OR "fall"))))) OR "BRAIN INJURIES, TRAUMATIC" [MeSH]) NOT "PSYCHOLOGICAL TRAUMA" [MeSH])

AND #3 (population)
("ADULT"[MeSH] OR "AGED"[MeSH] OR "geriatric*"[Tiab] OR "elderl*"[Tiab] OR "older adult*"[Tiab] OR "older patient*"[Tiab] OR "older man"[Tiab] OR "older men"[Tiab] OR "older male"[Tiab] OR "older woman"[Tiab] OR "older women"[Tiab] OR "older female"[Tiab] OR "older person*"[Tiab] OR "frail*"[Tiab] OR "senior*"[Tiab])

AND #4 (tool)
"OUTCOME ASSESSMENT, HEALTH CARE"[MeSH] OR "PATIENT OUTCOME ASSESSMENT"[MeSH] OR "QUALITY OF LIFE"[MeSH] OR ("patient reported outcome*" OR "patient-reported outcome*" OR "patient-related outcome*" OR "patient related outcome*") OR ((hrql OR hrqol OR qol OR ql OR "quality of life") AND (index OR indices OR instrument* OR measure* OR questionnaire* OR profile* OR scale* OR score* OR status OR survey*))

AND #5
((instrumentation[sh] OR “Validation Studies”[pt] OR "psychometrics"[MeSH] OR psychometr*[tiab] OR clinimetr*[tiab] OR clinometr*[tiab] OR "observer variation"[MeSH] OR “observer variation”[tiab] OR "discriminant analysis"[MeSH] OR reliability[tiab] OR validity[tiab] OR validate[tiab] OR validation[tiab] OR "internal consistency"[tiab] OR (cronbach*[tiab] AND (alpha[tiab] OR alphas[tiab])) OR "item correlation"[tiab] OR "item correlations"[tiab] OR "item selection"[tiab] OR "item selections"[tiab] OR "item reduction"[tiab] OR "item reductions"[tiab] OR precision[tiab] OR "sensitivity to change"[tiab] OR test–retest[tiab] OR (test[tiab] AND retest[tiab]) OR (reliab*[tiab] AND (test[tiab] OR retest[tiab])) OR stability[tiab] OR interrater[tiab] OR inter-rater[tiab] OR intrarater[tiab] OR intra-rater[tiab] OR intertester[tiab] OR inter-tester[tiab] OR intratester[tiab] OR intra-tester[tiab] OR interobserver[tiab] OR inter-observer[tiab] OR intraobserver[tiab] OR intra-observer[tiab] OR kappa[tiab] OR kappa’s[tiab] OR kappas[tiab] OR "coefficient of variation"[tiab] OR dimensionality[tiab] OR "multitrait scaling analysis"[tiab] OR "multitrait scaling analyses"[tiab] OR "item discriminant"[tiab]OR "interscale correlation"[tiab] OR "interscale correlations"[tiab] OR "individual variability"[tiab] OR "interval variability"[tiab] OR "rate variability"[tiab] OR "variability analysis"[tiab] OR "standard error of measurement"[tiab] OR (limit[tiab] AND detection[tiab]) OR interpretab*[tiab] OR (small*[tiab] AND (real[tiab] OR detectable[tiab]) AND (change[tiab] OR difference[tiab])) OR "meaningful change"[tiab] OR "minimal important change"[tiab] OR "minimal important difference"[tiab] OR "minimally important change"[tiab] OR "minimally important difference"[tiab] OR "minimal detectable change"[tiab] OR "minimal detectable difference"[tiab] OR "minimally detectable change"[tiab] OR "minimally detectable difference"[tiab] OR "minimal real change"[tiab] OR "minimal real difference"[tiab] OR "minimally real change"[tiab] OR "minimally real difference"[tiab] OR "ceiling effect"[tiab] OR "floor effect" [tiab] OR "Item response model"[tiab] OR IRT[tiab] OR Rasch[tiab] OR "Differential item functioning"[tiab] OR DIF[tiab] OR "computer adaptive testing"[tiab] OR "item bank"[tiab] OR "cross-cultural equivalence"[tiab]))

NOT #6
(("addresses"[Publication Type] OR "biography"[Publication Type] OR "case reports"[Publication Type] OR "comment"[Publication Type] OR "directory"[Publication Type] OR "editorial"[Publication Type] OR "festschrift"[Publication Type] OR "interview"[Publication Type] OR "lectures"[Publication Type] OR "legal cases"[Publication Type] OR "legislation"[Publication Type] OR "letter"[Publication Type] OR "news"[Publication Type] OR "newspaper article"[Publication Type] OR "patient education handout"[Publication Type] OR "popular works"[Publication Type] OR "congresses"[Publication Type] OR "consensus development conference"[Publication Type] OR "consensus development conference, nih"[Publication Type] OR "practice guideline"[Publication Type]) NOT ("animals"[MeSH Terms] NOT "humans"[MeSH Terms]))

CROM extraction

#1.1 Mortality, morbidity, complications, discharge
("Mortality" [MeSH] OR "mortality" [Subheading] OR "Hospital Mortality" [Mesh] OR "mortality registration" [Tiab] OR "mortality" [Tiab] OR "hospital mortality" [Tiab] OR "survival" [Tiab] OR "dead" [Tiab] OR "death" [Tiab] OR "loss of life" [Tiab] OR "cause of death" [Tiab] OR "trauma registry" [all fields] OR "Morbidity" [Mesh] OR "Morbidity" [all fields] OR "Failure to Rescue, Health Care"[Mesh] OR "complication*" [Tiab] OR "complications" [Subheading] OR "adverse effects" [Tiab] OR "Patient Discharge"[Mesh] OR "discharge" [tiab] OR "hospital discharge" [Tiab])
#1.2 Ambulatory function
("ambulatory function"[All Fields] OR "ability to walk"[Tiab] OR "walking ability"[Tiab] OR "walking proficiency"[Tiab] OR "ability to move"[Tiab] OR "ambulatory capacity"[Tiab] OR "ambulatory ability"[Tiab] OR "walking performance"[Tiab] Or "walking skill"[Tiab] OR "functional movement"[Tiab] OR "functional mobility"[Tiab] OR "walking capability"[Tiab] OR "walking function"[Tiab] OR "walking capacity"[Tiab] OR "walking"[Tiab])

#1.3 Sleep
("Sleep"[MeSH] OR "Sleep quality"[MeSH] OR "sleep" [Tiab] OR "sleeping"[Tiab] OR "sleep* disorder"[Tiab] OR "sleep* problems"[Tiab] OR "Pittsburgh Sleep Quality Index"[Tiab] OR "PSQI"[Tiab] OR "the Epworth Sleepiness Scale"[Tiab] OR "ESS"[Tiab])

#1.4 Sexual function
("Sexual Dysfunction, Physiological"[Mesh] OR "Sexual Health"[Mesh] OR "sexual function"[Tiab] OR "sex* functioning"[Tiab] OR "erectile function"[Tiab] OR "sexual performance"[Tiab] OR "erotic function"[Tiab] OR "sexual activity"[Tiab] OR "sexual health"[Tiab] OR "sexual response"[Tiab] OR "sexual dysfunction"[Tiab] OR "sexual disfunction"[Tiab])

#1.5 Autonomy
("Personal Autonomy"[Mesh] OR "autonomy"[Tiab] OR "self-determination"[Tiab] OR "self determination"[Tiab] OR "self-rule"[Tiab] OR "volition"[Tiab] OR "free will"[Tiab] OR "accord"[Tiab])

#1.6 Change in employment
("employment"[MeSH] OR "occupational change"[Tiab] OR "employment"[Tiab] OR "occupation"[Tiab] OR "change in employment"[Tiab] OR "change in occupation"[Tiab] OR "labor force"[Tiab] OR "employment termination"[Tiab] OR "employment status"[Tiab] OR "return to work"[Tiab] OR "unable to work"[Tiab])

AND #2
((WOUNDS AND INJURIES [MeSH] NOT "Asphyxia" [MeSH] NOT "Barotrauma" [MeSH] NOT "Birth injuries" [MeSH] NOT "Bites and Stings" [MeSH] NOT "Drowning" [MeSH] NOT "Heat Stress Disorders" [MeSH] NOT "Radiation injuries" [MeSH] NOT "Tooth injuries" [MeSH] OR "trauma" [Tiab] OR "ACUTE CARE SURGERY"[MeSH] OR ("ORTHOPEDICS" [MeSH] OR ("orthop*edics"[Tiab] adj3 ("trauma*" OR "polytrauma*" OR "multiple trauma*" OR (("serious*" OR "severe*" OR "major" OR "life threaten*") adj3 ("accident*" OR "injur*" OR "fall"))))) OR "BRAIN INJURIES, TRAUMATIC" [MeSH]) NOT "PSYCHOLOGICAL TRAUMA" [MeSH])

AND #3 (population)
("ADULT"[MeSH] OR "AGED"[MeSH] OR "geriatric*"[Tiab] OR "elderl*"[Tiab] OR "older adult*"[Tiab] OR "older patient*"[Tiab] OR "older man"[Tiab] OR "older men"[Tiab] OR "older male"[Tiab] OR "older

woman"[Tiab] OR "older women"[Tiab] OR "older female"[Tiab] OR "older person*"[Tiab] OR "frail*"[Tiab] OR "senior*"[Tiab] OR “adult” [Tiab])

AND #5
((instrumentation[sh] OR “Validation Studies”[pt] OR "psychometrics"[MeSH] OR psychometr*[tiab] OR clinimetr*[tiab] OR clinometr*[tiab] OR "observer variation"[MeSH] OR “observer variation”[tiab] OR "discriminant analysis"[MeSH] OR reliability[tiab] OR validity[tiab] OR validate[tiab] OR validation[tiab] OR "internal consistency"[tiab] OR (cronbach*[tiab] AND (alpha[tiab] OR alphas[tiab])) OR "item correlation"[tiab] OR "item correlations"[tiab] OR "item selection"[tiab] OR "item selections"[tiab] OR "item reduction"[tiab] OR "item reductions"[tiab] OR precision[tiab] OR "sensitivity to change"[tiab] OR test–retest[tiab] OR (test[tiab] AND retest[tiab]) OR (reliab*[tiab] AND (test[tiab] OR retest[tiab])) OR stability[tiab] OR interrater[tiab] OR inter-rater[tiab] OR intrarater[tiab] OR intra-rater[tiab] OR intertester[tiab] OR inter-tester[tiab] OR intratester[tiab] OR intra-tester[tiab] OR interobserver[tiab] OR inter-observer[tiab] OR intraobserver[tiab] OR intra-observer[tiab] OR kappa[tiab] OR kappa’s[tiab] OR kappas[tiab] OR "coefficient of variation"[tiab] OR dimensionality[tiab] OR "multitrait scaling analysis"[tiab] OR "multitrait scaling analyses"[tiab] OR "item discriminant"[tiab]OR "interscale correlation"[tiab] OR "interscale correlations"[tiab] OR "individual variability"[tiab] OR "interval variability"[tiab] OR "rate variability"[tiab] OR "variability analysis"[tiab] OR "standard error of measurement"[tiab] OR (limit[tiab] AND detection[tiab]) OR interpretab*[tiab] OR (small*[tiab] AND (real[tiab] OR detectable[tiab]) AND (change[tiab] OR difference[tiab])) OR "meaningful change"[tiab] OR "minimal important change"[tiab] OR "minimal important difference"[tiab] OR "minimally important change"[tiab] OR "minimally important difference"[tiab] OR "minimal detectable change"[tiab] OR "minimal detectable difference"[tiab] OR "minimally detectable change"[tiab] OR "minimally detectable difference"[tiab] OR "minimal real change"[tiab] OR "minimal real difference"[tiab] OR "minimally real change"[tiab] OR "minimally real difference"[tiab] OR "ceiling effect"[tiab] OR "floor effect" [tiab] OR "Item response model"[tiab] OR IRT[tiab] OR Rasch[tiab] OR "Differential item functioning"[tiab] OR DIF[tiab] OR "computer adaptive testing"[tiab] OR "item bank"[tiab] OR "cross-cultural equivalence"[tiab]) OR “registration” [Tiab] OR “registry” [Tiab] OR “recording” [Tiab] OR “record” [Tiab] OR “index” [Tiab] OR “indices” [Tiab] OR “instrument*” [Tiab] OR “measure*” [Tiab] OR “questionnaire*” [Tiab] OR “profile*” [Tiab] OR “scale*” [Tiab] OR “score*” [Tiab] OR “status” [Tiab] OR “survey*” [Tiab] OR “benchmark*” [all fields])

NOT #6
(("addresses"[Publication Type] OR "biography"[Publication Type] OR "case reports"[Publication Type] OR "comment"[Publication Type] OR "directory"[Publication Type] OR "editorial"[Publication Type] OR "festschrift"[Publication Type] OR "interview"[Publication Type] OR "lectures"[Publication Type] OR "legal cases"[Publication Type] OR "legislation"[Publication Type] OR "letter"[Publication Type] OR "news"[Publication Type] OR "newspaper article"[Publication Type] OR "patient education handout"[Publication Type] OR "popular works"[Publication Type] OR "congresses"[Publication Type] OR "consensus development conference"[Publication Type] OR "consensus development conference, nih"[Publication Type] OR "practice guideline"[Publication Type]) NOT ("animals"[MeSH Terms])

Supplementary material 3 (S3) – Overview ethics committees and regulatory bodies providing ethics approval or exemption for the patient survey

Country	Ethics committee or regulatory body	Ethics approval or exemption number
Australia	Monash University Human Research Ethics Committee	45934
The Netherlands	Medical Research Ethics Committee (MREC) NedMec	24-340/DB
New Zealand	Health and Disability Ethics Committees	Dec: 17/10/2024 10:12
United Kingdom	NHS Health Research Authority	Dec: 27/11/2024 16:12
United States	North Star Review board	NB400241

Supplementary material 4 (S4) – Baseline demographic characteristics of the Major Injury Working Group members and participants of the patient validation survey and professionals open review survey

Demographic characteristics of participants		Major Injury Working Group	Patient Validation Survey	Professional Open Review Survey
**Total participants**	% (n)	100% (n=28)	100% (n=121)	100% (n=70)
**Gender**	Female	39·3%	45·5%	N/R
	Male	60·7%	50·4%	N/R
	Prefer not to say	0·0%	4·1%	N/R
**Age (years)**	18-20	N/R	1·0%	N/R
	21-30	N/R	4·0%	N/R
	31-45	N/R	14·0%	N/R
	46-60	N/R	40·0%	N/R
	61-75	N/R	32·0%	N/R
	76-85	N/R	8·0%	N/R
	Prefer not to say	N/R	1·0%	N/R
**Type of patient experience**	Lived experience	21·4%	87·6%	N/A
	Carer	N/R	12·4%	N/A
**Type of personal and/or professional disorder experience**	Orthopaedic injuries	28·6%	36·1%	77·1%
	Traumatic injuries	50·0%		0·0%
	Spinal cord injuries	7·1%	16·1%	55·7%
	Traumatic brain injuries	14·3%	12·8%	60·0%
	Burn injuries	3·6%	4·4%	42·9%
	Other injuries	42·9%	20·0%	39·0%
	Prefer not to say	0·0%	2·2%	N/A
**Field of expertise**	Advocacy or charity professional	0·0%	N/A	1·4%
	Commercial or industry representative	0·0%	N/A	1·4%
	Government, policy or commissioning professional	0·0%	N/A	2·9%
	Health or social care practitioner (i.e. Clinician, allied health professional, social worker)	78·6%	N/A	77·1%
	Other professional representative not otherwise specified	0·0%	N/A	2·9%
	Researcher or educator	0·0%	N/A	14·3%
**Country**	Australia	14·3%	47·93%	32·9%
	USA	32·1%	25·62%	15·7%
	UK	10·7%	11·57%	10·0%
	New Zealand	7·1%	9·09%	15·7%
	Netherlands	14·3%	1·65%	1·4%
	Brazil	0·0%	0·0%	2·9%
	Canada	0·0%	0·0%	2·9%
	Ethiopia	0·0%	0·0%	1·4%
	Germany	0·0%	0·0%	4·3%
	India	0·0%	0·0%	4·3%
	Malaysia	0·0%	0·0%	1·4%
	Norway	0·0%	0·0%	1·4%
	Philippines	0·0%	0·0%	1·4%
	Saudi Arabia	3·6%	0·0%	1·4%

	South Africa	3·6%	0·0%	1·4%
	France	0·0%	0·0%	1·4%
	Sweden	3·6%	0·0%	0·0%
	Brazil	3·6%	0·0%	0·0%
	Japan	3·6%	0·0%	0·0%
	China	3·6%	0·0%	0·0%
	Other	0·0%	4·13%	0·0%
N/R = Not reported (not asked in respective survey or from working group				
N/A = Not applicable for the participants in the respective surveys				

Supplementary material 5 (S5) – Overview of psychometric properties of the PROMS selected by the Major Injury Working Group

Psychometric Properties	EQ-5D-5L	PROMIS GH-10	VR-12	SF-12	ASSIST-LITE	PC- PTSD	PROMIS Sleep Disturbance 8b	PROMIS Satisfaction with Sex Life	MPAI-4	SCIM-SR	BSHS-B
Trauma population	+	-	+	+	-	-	+	+	+	+	+
Sensitivity to change/ Responsiveness	+	?	?	=	?	?	+	+	?	?	=
Content Validity	=	+	+	?	+	+	+	+	?	+	+
Construct Validity	=	=	+	+	=	+	+	+	+	?	+
Discriminative validity	+	=	?	+	+	?	?	?	?	=	=
Test- retest reliability	+	+	?	+	?	+	+	+	+	=	?
Internal consistency/reliability	?	+	?	+	+	+	+	+	+	=	+
# Items	5	10	12	12	7	4	8	5	35	19	40
Time to complete	5-10 min	5 min	5-10 min	5 min	3-5 min	5 min	5 min	5 min	15 min	10-15 min	10 min
Age range	12+	18+	18+	18+	18+	18+	18+	18+	18+	18+	18+

Key

Conceptual & measurement model	Conceptual and measurement model*	Covers relevant outcome domain		Does not cover relevant outcome domain	No information available
	Target population	Validated for people with traumatic injuries		Not validated for people with traumatic injurie	
Licensing	Information on licensing and costs*	Tool available to use for free		High licensing costs	
Translation	Number of languages/translations*	≥ 3	Weak evidence	<3	
Validity	Sensitivity to change	Clear evidence	Weak evidence	No evidence	
	Content validity	Clear evidence	Weak evidence	No evidence	
	Construct validity	Clear evidence	Weak evidence	No evidence	
	Discriminant validity	Clear evidence	Weak evidence	No evidence	
Reliability	Test-retest reliability	r > 0.7	Weak evidence	r < 0.7 or no evidence	
	Internal consistency	α > 0.7	Weak evidence	α < 0.7 or no evidence	
Burden	Patient burden (time energy and literacy demand)				
	Administrative burden (clinical/administrative/investigator/data analyst burden)				
Age group coverage	Ability of the tool to cover more than one age group in scope				

Items marked with an asterisk (*) are not further elaborated in the psychometric properties, as these were identical across all PROMS and met the green criteria.
